# Genetic variation associated with adult migration timing in lineages of Steelhead and Chinook Salmon in the Columbia River

**DOI:** 10.1111/eva.13626

**Published:** 2023-12-28

**Authors:** Shawn R. Narum, Rebekah Horn, Stuart Willis, Ilana Koch, Jon Hess

**Affiliations:** ^1^ Columbia River Inter‐Tribal Fish Commission Hagerman Genetics Laboratory Hagerman Idaho USA; ^2^ Columbia River Inter‐Tribal Fish Commission Portland Oregon USA

**Keywords:** GREB1L/ROCK1, interior lineages, migration timing, salmonids

## Abstract

With the discovery of a major effect region (GREB1L, ROCK1) for adult migration timing in genomes of both Chinook Salmon and Steelhead, several subsequent studies have investigated the effect size and distribution of early and late migration alleles among populations in the Columbia River. Here, we synthesize the results of these studies for the major lineages of Chinook Salmon and Steelhead that include highly distinct groups in the interior Columbia River that exhibit atypical life histories from most coastal lineage populations of these two species. Whole‐genome studies with high marker density have provided extensive insight into SNPs most associated with adult migration timing, and suites of markers for each species have been genotyped in large numbers of individuals to further validate phenotypic effects. For Steelhead, the largest phenotypic effect sizes have been observed in the coastal lineage (36% of variation for passage timing at Bonneville Dam; 43% of variation for tributary arrival timing) compared to the inland lineage (7.5% of variation for passage timing at Bonneville Dam; 8.4% of variation for tributary arrival timing) that overwinter in freshwater prior to spawning. For Chinook Salmon, large effect sizes have been observed in all three lineages for multiple adult migration phenotypes (Coastal lineage: percentage of variation of 27.9% for passage timing at Bonneville Dam, 28.7% for arrival timing for spawning; Interior ocean type: percentage of variation of 47.6% for passage timing at Bonneville Dam, 39.6% for tributary arrival timing, 77.9% for arrival timing for spawning; Interior stream type: percentage of variation of 35.3% for passage at Bonneville Dam, 9.8% for tributary arrival timing, 4.7% for arrival timing for spawning). Together, these results have extended our understanding of genetic variation associated with life history diversity in distinct populations of the Columbia River, however, much research remains necessary to determine the causal mechanism for this major effect region on migration timing in these species.

## 1 INTRODUCTION

Pacific salmonids have distinct adult migration timing within and among populations that is well known to be highly heritable (e.g., Carlson & Seamons, 2008), and variation for this trait is particularly notable among runs of Steelhead (*Oncorhynchus mykiss*; Busby et al., 1996) and Chinook Salmon (*Oncorhynchus tshawytscha*; Myers et al., 1998) in the Pacific Northwest (PNW) of N. America. In coastal populations, Steelhead are typically considered either winter or summer run depending on the timing of their return to freshwater (Busby et al., 1996), while Chinook Salmon are characterized as either spring or fall run (Myers et al., 1998). However, patterns in adult migration timing differ in lineages of both species that migrate to the interior Columbia River, with more constricted run‐timing due to the long migration distance populations must travel to return to spawning grounds (Quinn et al., 2016). In regions such as the interior Columbia River, populations of inland lineage Steelhead only return in summer through fall months and overwinter in freshwater (Hess, Zendt, et al., 2016; Quinn et al., 2016; Keefer, Boggs, et al., 2008; Keefer, Caudill, et al., 2008), while the two interior lineages of Chinook Salmon return to freshwater with a narrow range of return timing either as summer/fall (interior ocean‐type lineage) or spring/summer (interior stream‐type lineage) (Hess et al., 2014; Keefer et al., 2004; Narum et al., 2010). Several studies in recent years have identified a major effect locus for adult migration timing in both Steelhead and Chinook Salmon (reviewed in Waples et al., 2022). Initial studies used reduced representation sequencing methods to identify three SNPs in the GREB1L gene region that were strongly associated with summer vs. winter migration timing in Steelhead (Hess, Zendt, et al., 2016; Prince et al., 2017) and spring vs. fall migration timing in coastal populations of Chinook Salmon (Prince et al., 2017). Subsequent studies applied whole‐genome sequencing methods resulting in much higher marker density that elucidated strong patterns of association for SNPs within a single region spanning GREB1L through the adjacent gene ROCK1, with especially strong signals observed in the intergenic region for both Steelhead and Chinook Salmon (Micheletti, Hess, et al., 2018; Narum et al., 2018; Thompson et al., 2019). A subsequent study with individual whole‐genome sequences of Chinook Salmon from coastal populations (Thompson et al., 2020) revealed structural variation in the form of duplication in this intergenic region that may be responsible for high linkage disequilibrium and strong association with migration timing. Further, this genetic region was directly associated with adult migration timing in coastal populations of Chinook Salmon (Thompson et al., 2020). However, the pattern of association has been shown to be much more complex for lineages of Steelhead (Willis et al., 2020) and Chinook Salmon (Willis et al., 2021) migrating to the interior Columbia River. This complexity for interior lineages of the Columbia River was also a noted contrast from coastal lineages in a recent review of this major effect gene region (Waples et al., 2022). While efforts have been made to refine understanding of the causal region for adult migration timing in both Steelhead and Chinook Salmon, it is important to note that the inferred strength of phenotypic effects of different SNPs in a genomic region arises not only from real biological effects but is also influenced by historical recombination events and sampling effects that can vary across studies. Thus, the causal variation is not likely to be directly detected with association testing alone.

Here, we synthesize the complex patterns of association within the major effect region on Chromosome 28 (spanning GREB1L to ROCK1) for lineages of Steelhead (Omy28) and Chinook Salmon (Ots28) in the interior Columbia River relative to coastal lineages of each species. Further context is also provided for (1) SNP markers that have been developed from this region for validation and resolving detail of associated run‐timing phenotypes in Steelhead and Chinook Salmon, (2) conservation priorities for each species in the Columbia River (Box 1), and (3) future directions for ongoing research related to GREB1L/ROCK1 and migration timing in salmonids.

BOX 1 **Are Early Migrating Steelhead and Chinook Salmon of Conservation Concern in the Columbia River?**

Until the discovery of the major effect genes GREB1L/ROCK1 for migration timing, the prevailing view was that early migrating Chinook Salmon and Steelhead, which have declined disproportionately over the last several decades (Gustafson et al., 2007; Thurow et al., 2020), could be recovered from standing genetic variation within a population (reviewed by Waples et al., 2022). However, new studies have illustrated that the loss of early alleles at GREB1L/ROCK1 within a population cannot be recovered once lost from a population (Thompson et al., 2019), except through an influx of early alleles from outside populations. This has led to further scrutiny of how early migrating Chinook Salmon and Steelhead are designated within the same ESU/DPS along with late migrating fish. In some coastal rivers, regional conservation units have been established to specifically protect early migrating Chinook Salmon and Steelhead (reviewed in Waples et al., 2022). However, the interior lineages of both species in the Columbia River are highly distinct across the genome from coastal lineage Chinook Salmon and Steelhead, but early and late migrating individuals are represented within all lineages and require conservation. Thus, questions remain regarding conservation concerns for early migrating Chinook Salmon and Steelhead in the interior Columbia River. This box provides a brief perspective on conservation concerns for each lineage. Specific DPS are mentioned where relevant, but comments are not comprehensive of all designated DPS within the Columbia River Basin.

**Steelhead**

- *Coastal lineage*
  Summer‐run Steelhead, with early alleles that have a major effect on run‐timing, are more rare than winter run in most coastal rivers including those in the lower Columbia River (Table 1). This has led to regional conservation listings and specific protection for summer‐run steelhead in coastal populations as reviewed by Waples et al. (2022), but early returning steelhead are not currently designated as separate conservation units. There have been multiple petitions for separate DPS designation of early migrating populations of Steelhead in coastal rivers outside of the Columbia River Basin, but federal agencies have currently retained designation of units that include both early and late components that are considered necessary for a DPS/ESU to be viable (Waples et al., 2022).
- *Inland lineage*
  Early migration alleles are rare in the inland lineage of steelhead in the Columbia River (Table 1), but the association and effect on migration timing are not as strong in the inland lineage as in the coastal lineage (Willis et al., 2020). This is largely due to the overwintering behavior that is displayed by most inland Steelhead, but inland Steelhead continues to demonstrate bimodal migration patterns passing Bonneville Dam and tributary arrival timing (see Figure 2) that have yet to be fully resolved with variation at GREB1L/ROCK1. Thus, further studies are needed to validate whether complex individual phenotypes in inland Steelhead are explained by GREB1L/ROCK1 or additional regions of the genome that warrant special conservation measures. Further, populations near the crest of the Cascade Mountains appear intermediate to the two major lineages and need special consideration since they appear to experience high levels of gene flow with both coastal and inland lineage fish. These studies are needed to provide direction for conservation units and listing under the ESA.

**Chinook salmon**

- *Coastal lineage*
  Spring‐run Chinook Salmon with early alleles are more rare than fall run fish with late alleles in coastal populations of the Columbia River (Table 2), except in the Willamette R. where fall run fish were not historically common due to exclusionary flows at Willamette Falls. This has led to regional conservation listings and specific protection for spring‐run Chinook Salmon as reviewed by Waples et al. (2022), but early returning Chinook Salmon are not currently designated as separate conservation units. There have been multiple petitions for separate DPS designation of early migrating populations of Chinook Salmon in coastal rivers outside of the Columbia River Basin, but federal agencies have currently retained the designation of units that include both early and late components that are considered necessary for a DPS/ESU to be viable (reviewed in Waples et al., 2022).
- *Interior ocean‐type lineage*
  Chinook Salmon with early alleles are more rare than those with late alleles in this interior lineage of the Columbia River (Table 2), and the effect size of alleles at GREB1L/ROCK1 is one of the strongest in any of the lineages of Chinook Salmon that have been evaluated. Further, haplotypes from each of the linkage blocks likely reflect a combination of adult migratory phenotypes (arrival for spawning, Koch & Narum, 2020; passage timing at Bonneville Dam and tributary arrival timing, Willis et al., 2021). In the upper Columbia River summer/fall ESU, the summer run component (early) is included within the same ESU as fall run (late) and recognizes that the summer run is rare and warrants specific conservation measures. The Snake River Fall ESU only recognizes Chinook salmon that return in fall, but fish with relatively early passage timing are represented in small numbers at Bonneville Dam (e.g., Hess et al., 2023) and early alleles have been detected in fish passing Lower Granite Dam and in the hatchery stock in the Snake River. Early returning fish from this lineage are rare in the Snake River but were likely present historically in the Clearwater River prior to extirpation (Holmes, 1961) and could be reintroduced to the system with a focus on stocks containing high frequency of early migrating alleles from this lineage.
- *Interior stream‐type lineage*
  While early alleles are predominant in the interior stream‐type lineage of Chinook Salmon (Table 2, Figure 14), this entire lineage remains of high conservation concern with populations expected to be extirpated in future decades (e.g., Bowerman et al., 2021; Crozier et al., 2021; Thurow et al., 2020). Even though Chinook Salmon in this lineage generally return early, there is a strong association and effect of alleles at GREB1L/ROCK1 on migration timing (Figure 14c). However, early migrating fish within this lineage do not appear to warrant specific conservation concerns since they are the most common. In contrast, late migrating alleles are rare in this lineage and are largely represented in populations returning to the Salmon River drainage. Fish from these late migrating populations with higher frequency of late alleles warrant special attention for conservation since they encounter more extreme environmental conditions in the migration corridors of the Columbia and Snake rivers and also experience higher rates of harvest in the Summer Management Period compared with early migrating counterparts that migrate in the Spring Management Period.

For purposes of this review, we define various specific phenotypes for adult migration timing (a.k.a., run‐timing) in Steelhead and Chinook Salmon as follows: *freshwater entry timing* is the date when anadromous salmonids enter freshwater from the ocean, as measured by day of entry into the mouth of the initial river of their migratory route; *passage timing at Bonneville Dam* (specific to the Columbia River) is the date of migration recorded for fish at a fixed location 234 km upstream of the mouth of the mainstem Columbia River, as measured by recovery of a PIT (passive integrated transponder) tag signal at the array or physical capture at the Adult Fish Facility at Bonneville Dam; *tributary arrival timing* is the date when fish enter the tributary of river system where they will attempt to spawn, as measured by a tag recovery or physical capture of individuals at the tributary confluence; *arrival timing to spawning grounds* is the date when fish that have been holding in a freshwater tributary arrive to the specific area where they attempt to spawn, as measured by tag recovery or physical capture near the location of redds; *spawn timing* is the date when mating events occur and gametes are deposited in redds, as measured by direct observation of individual spawning events and recovery of associated mating groups that contributed gametes. While previous studies have used the terminology “premature” and “mature” from Quinn et al. (2016) based on general migration phenology in Pacific salmonids, we avoid the use of these terms in this review since they are based on assumptions of maturation status that do not consistently relate to a specific adult migration timing phenotype. In place of premature and mature, the terms “early” and “late,” respectively, are applied to describe bimodal timing for each of the adult migration phenotypes following Ford et al. (2020). While the timing of various adult migration phenotypes may appear bimodal in some instances, treating adult migration timing traits as continuous variables may allow for more extensive statistical testing compared with the treatment of binary traits (e.g., Willis et al., 2021). Further, there are several additional phenotypes of interest for adult migration timing and behavior that include time of travel between tag recovery locations, duration spent in migration corridors, utilization of cool water refuges (tributary “dip‐in”), instances of indirect homing behavior (“overshoot” or “fallback”), or straying to nonnatal tributaries to spawn (Keefer, Boggs, et al., 2008; Keefer, Caudill, et al., 2008; Willis et al., 2020, 2021).

## 2 STEELHEAD OF THE COLUMBIA RIVER—SYNTHESIS OF PATTERNS OF GENOMIC ASSOCIATION WITH MIGRATION TIMING

Several salmonid species in the Columbia River basin have notable genetic divergence between populations in the interior region (east of the Cascade Mountains) relative to those in coastal streams due to geological features that have isolated lineages within species over evolutionary time (e.g., Waples et al., 2008) and are thus considered distinct lineages or subspecies (Behnke, 1992; Quinn, 2018). Native *O. mykiss* are broadly distributed throughout western N. America, with some remaining exclusively resident in freshwater (often referred to as Rainbow Trout or Redband Trout), while others exhibit anadromous migration and are called Steelhead. In Steelhead, several studies have identified two major lineages (e.g., Utter & Allendorf, 1977; Brannon et al., 2004; Blankenship et al., 2011; Quinn, 2018) that are considered subspecies representing a coastal lineage (*O. mykiss irideus*) and an inland lineage (*O. mykiss gairdneri*) within the Columbia River. Several populations comprise each of these major lineages, with a metapopulation structure that has led to the designation of five distinct population segments (DPS) (Busby et al., 1996; Waples et al., 2001). All five of the Steelhead DPS in the Columbia River are listed under the Endangered Species Act (ESA), with one Endangered (Upper Columbia River DPS), and the other four Threatened (Lower Columbia River, Middle Columbia River, Upper Willamette River, Snake River). Migration distance is one of the most substantial differences between coastal and inland lineages of *O. mykiss*, with evidence supporting local adaptation through environmental drivers of selection both in natal sites and through the migration corridor (e.g., Micheletti, Matala, et al., 2018).

**FIGURE 1:**
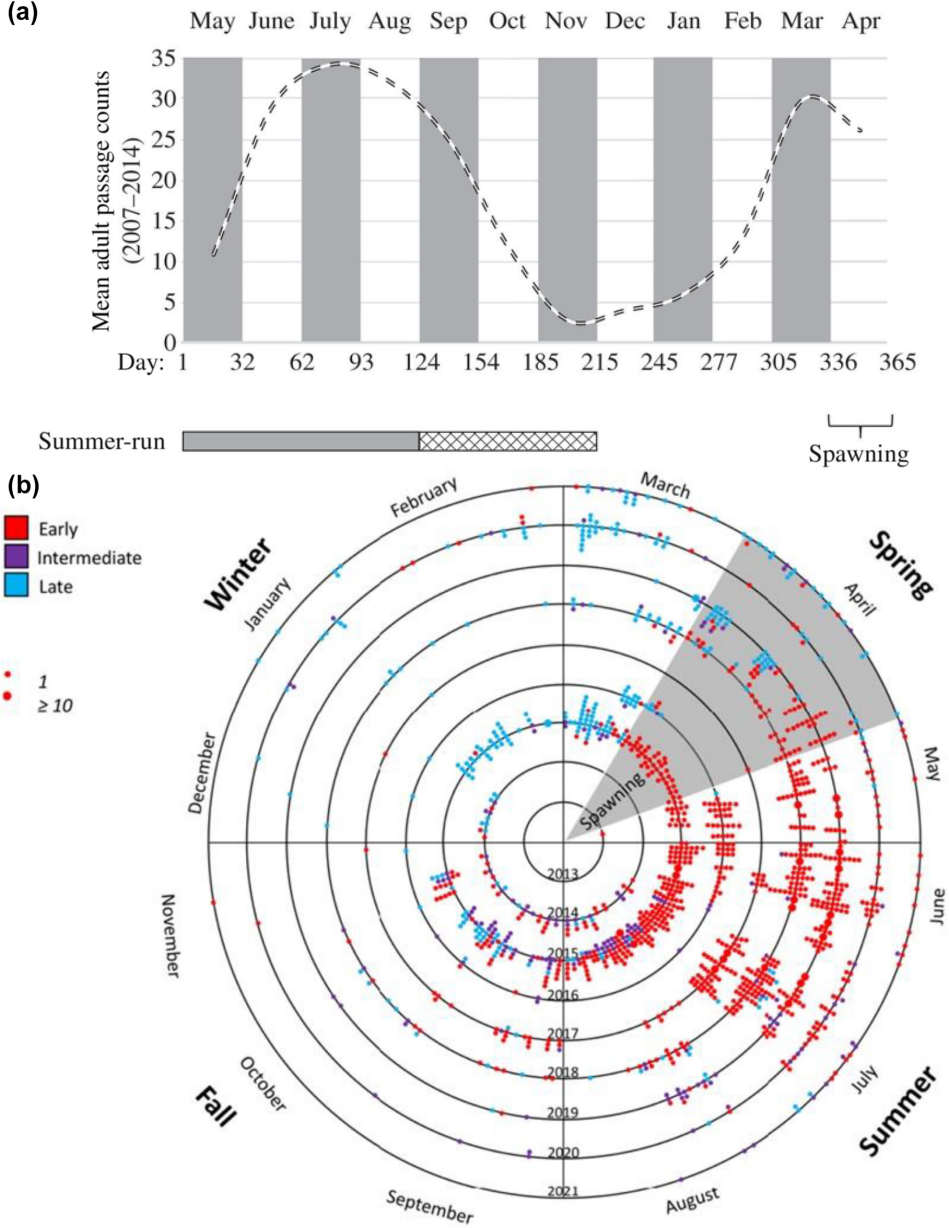
Illustration of typical coastal Steelhead run‐timing (data for Klickitat River population). (a) Adult migration timing is typically bimodal for coastal lineage populations that reflect summer versus winter run‐timing, with fish spawning in spring months (Hess, Zendt, et al., 2016). The dashed line represents the mean monthly counts of adult Steelhead passing Lyle Falls during 2007–2014. Ordinal “day” was reordered to begin with the summer‐run period and end after the spawning period. The bimodal distribution represents the peaks of the summer‐ and winter‐run Steelhead migration periods (horizontal bars) and includes a “transitional” period of overlap in distributions (hatched bars). (b) Despite bimodal patterns, individual Steelhead in the Klickitat River migrate all year round with the earliest (homozygous early genotypes [red] returning up to 1 year before spawning), intermediate timing for fish with heterozygous genotypes (purple), and late timing shortly before spawning (homozygous late genotypes in blue; Collins et al., 2023). The figure represents seasonal migration timing for individual adult Steelheads captured at the Lyle Falls trap in the lower Klickitat River between 2013 and 2021. Black rings of the circle represent Steelhead migrations for each sample year. The approximate spawning season in upstream tributaries is shown in the gray‐shaded piece of spring, but migration timing of Steelhead is shown as observed earlier at the trap located downstream at Lyle Falls. Each point is positioned according to the date each adult Steelhead returned to the Klickitat River. The size of each dot reflects the number of samples for a given date.

**FIGURE 2:**
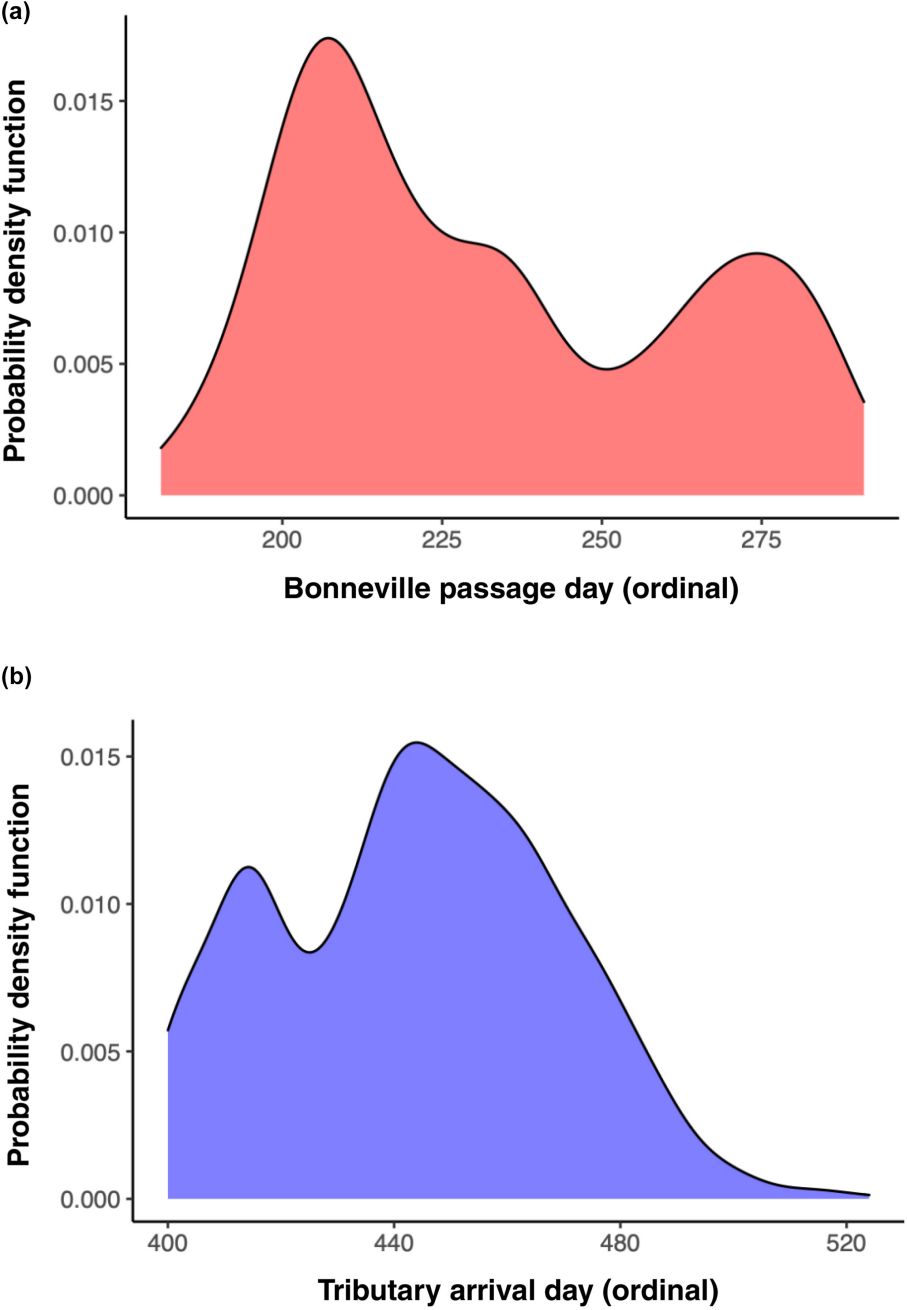
Illustration of Inland lineage Steelhead run‐timing at different stages of their adult migration (data represent PIT‐tagged fish from multiple populations). (a) Inland Steelhead passing Bonneville Dam has multi‐modal migration timing with a narrow range relative to coastal lineage fish which return in all months of the year (Willis et al., 2020). (b) Inland lineage Steelhead overwinter in freshwater before migrating at various times in the following months to spawning grounds (Willis et al., 2020).

Adult migration timing differs substantially between lineages of *O. mykiss* with coastal populations comprised of runs of summer and winter Steelhead that are typical of many coastal systems across the PNW from California to British Columbia of N. America (e.g., Busby et al., 1996; Figure 1 reproduced from Hess, Zendt, et al., 2016), while Steelhead of the inland lineage of the Columbia River return with constricted run‐timing due to the long migration distance populations must travel to return to spawning grounds (Quinn et al., 2016). Similarly, Steelhead populations in the interior Fraser River of British Columbia have distinct migration characteristics from those in coastal systems (as determined by radio telemetry; Renn et al., 2005). In regions such as the interior Columbia River, populations of inland lineage Steelhead only return in summer through fall months and overwinter in freshwater (Quinn et al., 2016; Keefer, Boggs, et al., 2008; Keefer, Caudill, et al., 2008; Hess, Zendt, et al., 2016; Figure 2). Thus, the coastal lineage of Steelhead displays typical adult migration patterns found across much of the coastal region of North America, while the inland lineage has distinct adult migration patterns relative to most other Steelhead populations in the PNW (Busby et al., 1996; Quinn et al., 2016).

Population genetic studies over past decades have consistently found limited neutral genetic differentiation between Steelhead with distinct migration timing regardless of marker type, which suggested high gene flow and interbreeding of summer and winter run fish within populations (e.g., allozymes, Chilcote et al., 1980; microsatellites, Narum et al., 2006; SNPs, Arciniega et al., 2016). However, more recent studies have implemented genome‐wide approaches to investigate the genetic basis for adult migration timing in Steelhead, which has been shown to be highly heritable (e.g., Carlson & Seamons, 2008). The first studies used reduced representation sequencing to survey the genome for variation associated with adult migration timing and successfully identified a common region near the GREB1L gene of Chromosome 28 (Hess, Zendt, et al., 2016; Prince et al., 2017). Despite low marker density that covered <1% of the genome, three SNP markers from the GREB1L region were found in both studies that were associated with summer (early) vs. winter (late) migrating Steelhead in the Klickitat River (Hess, Zendt, et al., 2016) and multiple populations from coastal rivers of California and Oregon (Prince et al., 2017). While these three SNPs were replicated in both studies and indicated strong support for GREB1L as a candidate gene region, there was a need for fine‐scale mapping with higher marker density to further pinpoint patterns of association with adult migration timing.

**FIGURE 3:**
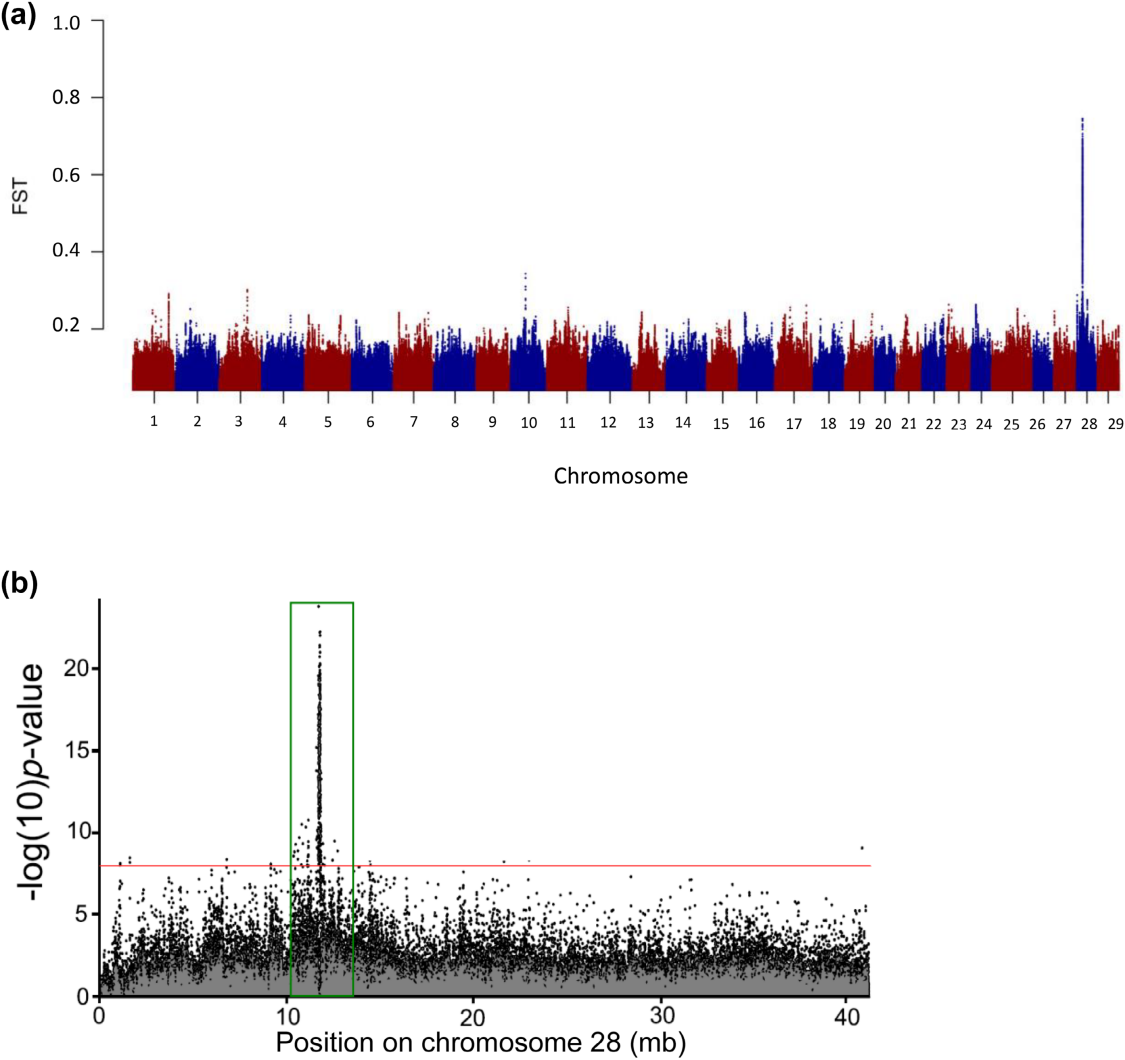
Genome scan with whole‐genome resequencing of summer versus winter run Steelhead from replicates of populations in the Klickitat and Kalama rivers. (a) Manhattan plot of genetic divergence of summer vs. winter run Steelhead across the *O. mykiss* genome, with the most substantial peak of FST at Chromosome 28 (Omy28; Micheletti, Hess, et al., 2018). (b) Peak of divergence (highlighted in green box) within Omy28 illustrating hundreds of closely located SNPs (Micheletti, Hess, et al., 2018).

A subsequent study implemented whole‐genome resequencing between replicate pairs of summer vs. winter Steelhead from two populations in the coastal lineage to enable fine‐scale mapping for SNPs associated with adult migration timing (Micheletti, Hess, et al., 2018). This study applied a standard Pool‐Seq approach (e.g., Schlötterer et al., 2014) that enabled 68.2% of the genome to be sequenced across phenotypic groups at an average of 33× coverage. Over 5 million SNP markers were tested for differences in allele frequency in the replicate pairs of summer vs. winter run Steelhead and identified a major peak of divergence on Omy28 with several 100 statistically significant markers near the GREB1L region (Figure 3, reproduced from Micheletti, Hess, et al., 2018). These markers spanned a region over 110 kb on Omy28, from GREB1L to an adjacent gene ROCK1 along with the intergenic region. The majority of markers occurred in the GREB1L region, but also several highly statistically significant SNPs in the intergenic region. While additional SNPs were statistically significant within nearby genes (several within ROCK1), these were considered less compelling candidates than the primary signal observed within GREB1L and the upstream regulatory region. This high‐density genome scan provided SNP targets for further validation, but this study also measured a greater level of detail of phenotypic variation for adult migration timing such as tributary arrival timing, along with passage timing at Bonneville Dam since these may be independent migration phenotypes for inland lineage Steelhead that overwinter in freshwater before spawning the following spring.

**FIGURE 4:**
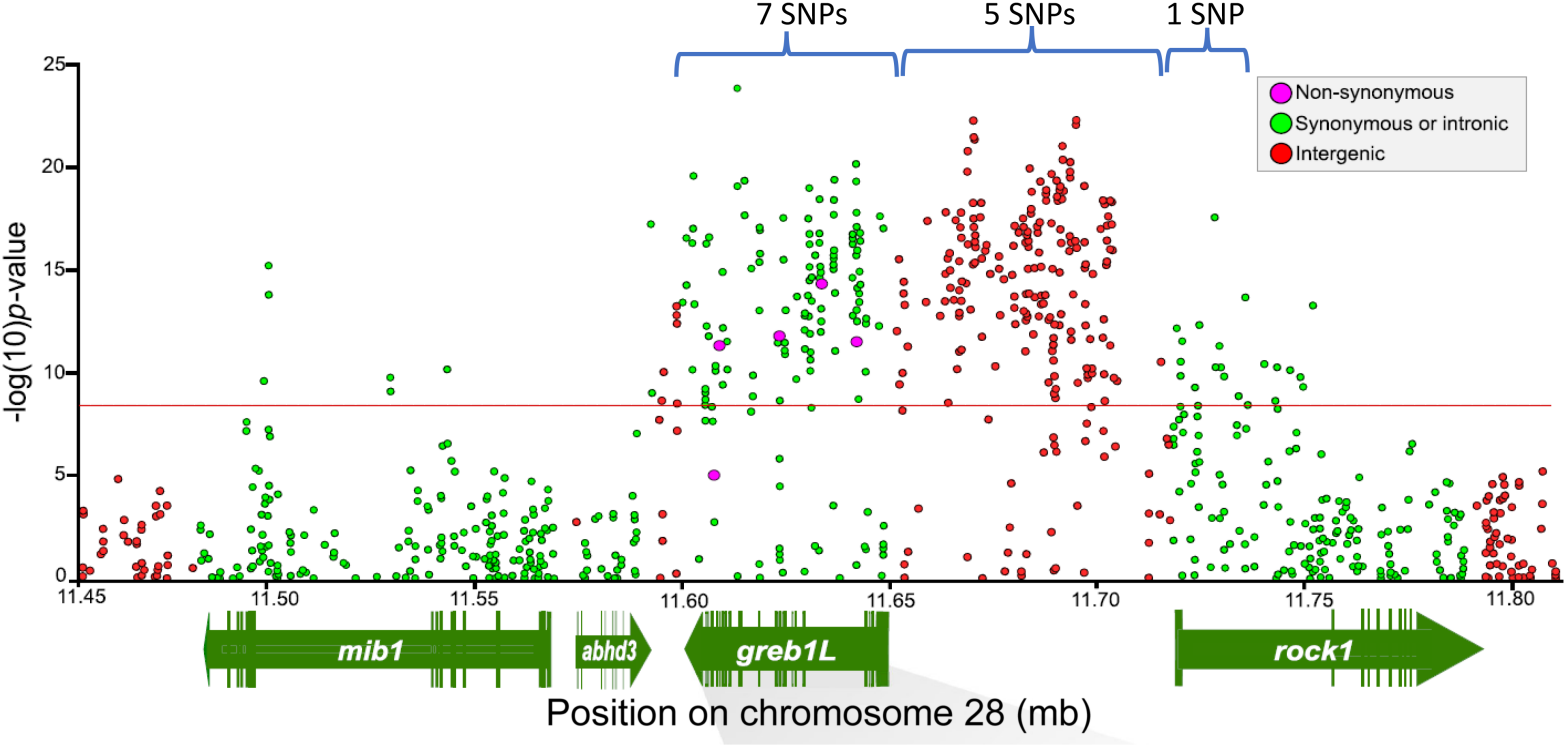
Development of candidate SNPs for adult migration timing for genotyping in GT‐seq panels for Steelhead. A total of 13 of the most statistically significant SNPs were designed for high throughput genotyping assays with 7 SNPs from the GREB1L region, 5 from intergenic, and 1 from ROCK1.

The most statistically significant SNP markers from Micheletti, Hess, et al. (2018) were selected for development into amplicon sequencing assays to enable validation testing with independent samples and multiple run‐timing phenotypes from large numbers of Steelhead. Phenotypes for individuals were based on collection or passage date at Bonneville Dam, and PIT‐tagged fish were tracked at arrays within tributaries to estimate tributary arrival timing. A total of 13 SNP markers were successfully incorporated into GT‐seq (genotyping thousands by sequencing) panels for this species (Figure 4, reproduced from Micheletti, Hess, et al., 2018), with 7 SNPs in the GREB1L region, 5 SNPs in the intergenic region, and 1 SNP in the ROCK1 region (Collins et al., 2020; Willis et al., 2020). Primers and probes for these 13 markers, and the key for early vs. late alleles in Steelhead, are provided in Table S1 (reproduced from Collins et al., 2020; Willis et al., 2020).

Two follow‐up studies provided validation for patterns of association with adult migration timing in Steelhead, along with evaluating the distribution and frequency of early alleles throughout the Columbia River basin (Collins et al., 2020; Willis et al., 2020). Both studies revealed distinct patterns between coastal and inland lineages related to their association with adult migration timing.

**FIGURE 5:**
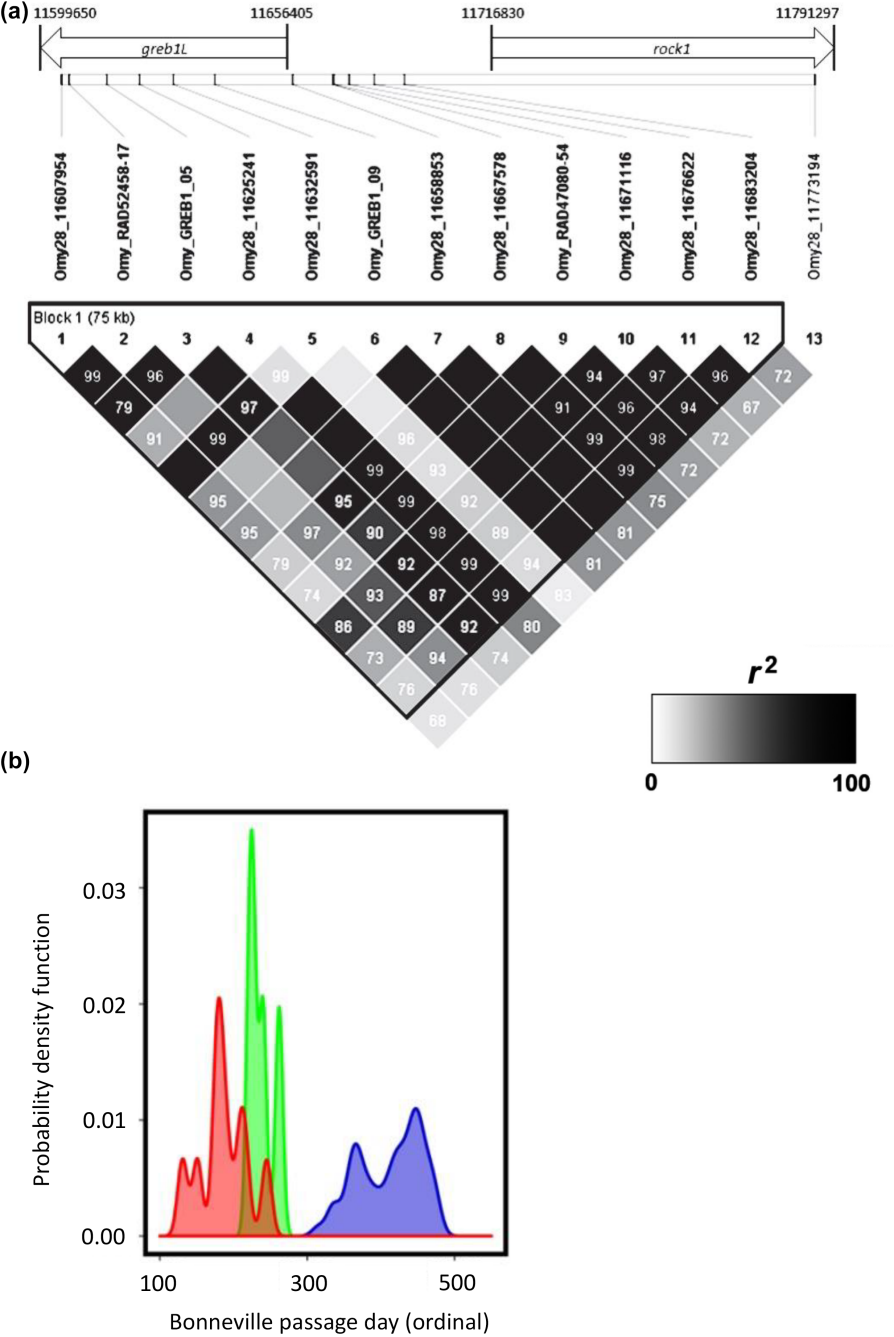
Coastal Steelhead linkage and association. (a) A single linkage block occurs for GREB1L/intergenic Omy28 in Coastal Steelhead from the Columbia River (Collins et al., 2020). Values within each block are *r* ^2^ values and represent a grayscale continuum from *r* ^2^ = 0 (light gray) through *r* ^2^ = 100 (black boxes). (b) Timing of Steelhead migration passage timing over Bonneville Dam based on individual genotypes for homozygous early (red), heterozygous (green), or homozygous late (blue; Willis et al., 2020).

In the coastal lineage, clear patterns of association with summer vs. winter adult migration timing were observed (Willis et al., 2020) along with support for a single linkage block for this lineage (Figure 5a; reproduced from Collins et al., 2020; Willis et al., 2020). For coastal lineage Steelhead with typical summer vs. winter run‐timing, haplotypes from this region were estimated to have a major effect on adult migration timing phenotypes (36% of variation for Bonneville passage timing; 43% of variation for tributary arrival timing; Willis et al., 2020). Heterozygous individuals also had intermediate return timing relative to early (summer) and late (winter) homozygous fish, but with greater overlap with early returning fish suggesting either additive inheritance or partial dominance of the early allele (Figure 5b, reproduced from Willis et al., 2020). Phenotypes from PIT‐tagged fish revealed high correlation (*r* ^2^ = 0.93) between passage timing at Bonneville Dam and tributary arrival timing for the coastal lineage, which suggests these two traits are not independent within this lineage or are exhibited jointly on a consistent basis. Finally, both studies (Collins et al., 2020; Willis et al., 2020) found that a hatchery stock known as Skamania strain was fixed for early alleles due to artificial selection for broodstock with extreme early return timing that initiated several decades ago (Ayerst, 1977) and is reflected in current migration patterns of this stock of Steelhead relative to others in the Columbia River (Hess, Ackerman, et al., 2016). Of 113 populations examined by Collins et al. (2020), 33 were considered to be either coastal lineage or “intermediate” between the two major lineages (near the crest of the Cascade Mountains). The Skamania strain was the only collection where early migration alleles were fixed in GREB1L region markers, and only seven others considered to be summer run had higher frequency of early migration haplotypes compared to higher frequency of late haplotypes in all the remaining populations (Collins et al., 2020).

**FIGURE 6:**
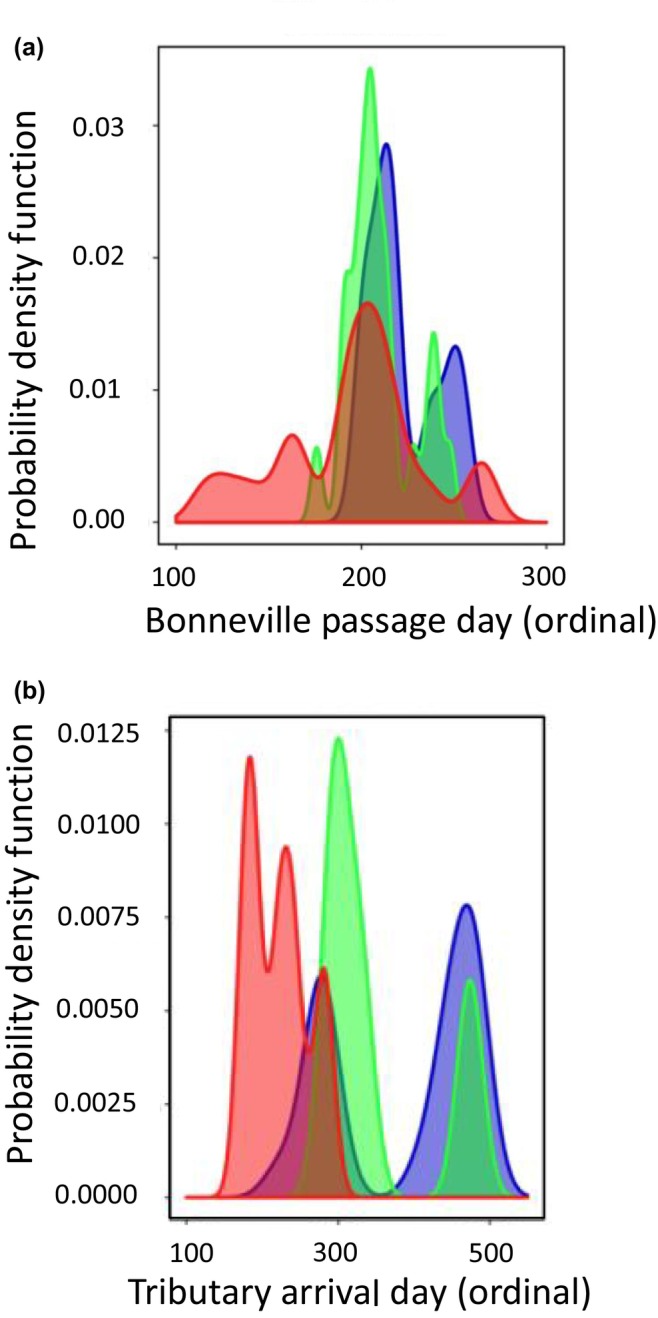
Contrasting patterns in association for the Wenatchee population of Inland Steelhead with different adult migration phenotypes based on individual genotypes for homozygous early (red), heterozygous (green), or homozygous late (blue; Willis et al., 2020). (a) Genotype patterns for passage timing at Bonneville Dam. (b) Genotype patterns for tributary arrival timing in the Wenatchee River.

In the inland lineage of Steelhead, patterns of association with GREB1L markers were more complex due to the distinct phenotypic variation of adult migration timing along with distinct genomic variation in the GREB1L/intergenic region for this lineage. Since inland Steelhead migrate to freshwater in summer through fall months and then overwinter in mainstem rivers before spawning in tributaries the following spring, many populations do not exhibit early versus late patterns of migration until arriving at spawning grounds (Micheletti, Zendt, et al., 2018; Willis et al., 2020). Thus, patterns of association were highly variable across populations of inland Steelhead and not consistent with regard to phenotypes of passage timing at Bonneville Dam or tributary arrival timing (Figure 6; reproduced from Micheletti, Zendt, et al., 2018; Willis et al., 2020). The effect size on adult migration timing was lower for inland populations (7.5% of variation for Bonneville passage timing; 8.4% of variation for tributary arrival timing) with these complex phenotypes relative to coastal populations (36% of variation for Bonneville passage timing; 43% of variation for tributary arrival timing; Willis et al., 2020), and heterozygous individuals had a broad range of return timing (Willis et al., 2020).

**FIGURE 7:**
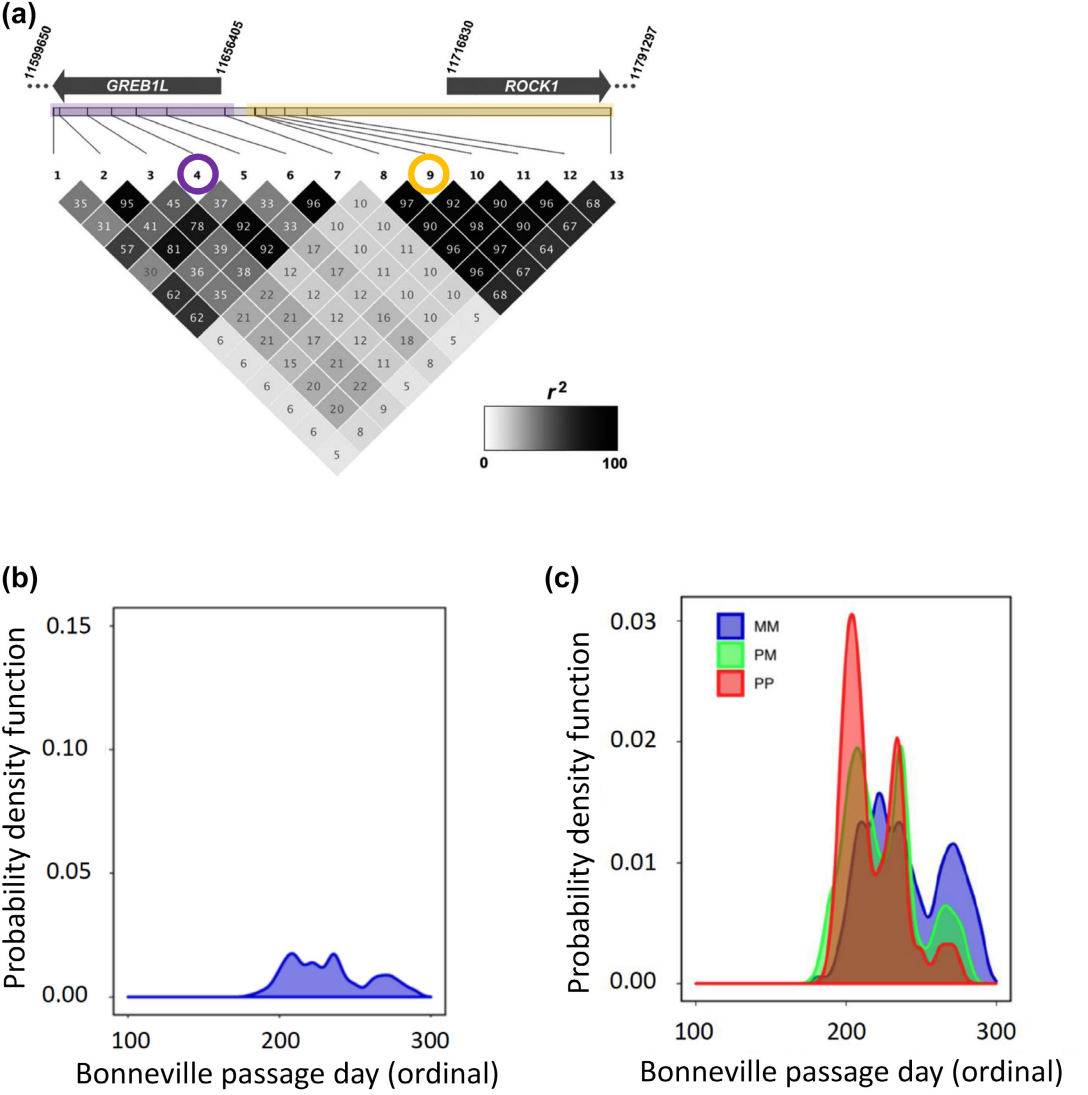
Linkage and association for Inland Steelhead from each of the two linkage blocks based on individual genotypes for homozygous early (red), heterozygous (green), or homozygous late (blue; Willis et al., 2020). (a) Two linkage blocks occur for GREB1L (purple) and intergenic (gold) for Omy28 in Inland Steelhead from the Columbia River (Willis et al., 2020). Values within each block are *r* ^2^ values and represent a grayscale continuum from *r* ^2^ = 0 (light gay) through *r* ^2^ = 100 (black boxes). (b) Genotype patterns for marker 4 (circled in purple; Figure 7a) from the GREB1L linkage block for Steelhead timing of passage at Bonneville Dam. (c) Genotype patterns for marker 9 (circled in gold; Figure 7a) from the intergenic/ROCK1 linkage block for Steelhead timing of passage at Bonneville Dam.

An additional complication for inland lineage Steelhead is that studies have found support for two distinct linkage blocks for markers within the GREB1L region versus those in the intergenic region (Figure 7a; reproduced from Collins et al., 2020; Willis et al., 2020), which makes evaluation of haplotypes more complex across the entire region of significance on Omy28. Further, SNP markers in the GREB1L region have very little variation relative to higher variation in the intergenic region in inland populations of Steelhead (Figure 7b, c; reproduced from Willis et al., 2020). This lack of variation could be due to a causal allele in the GREB1L region that conferred early return timing going to fixation in this lineage via positive selection, especially if the GREB1L region specifically effects freshwater entry timing in the inland lineage that exclusively enters the Columbia River several months prior to spawning the following spring. Phenotypes from PIT‐tagged fish revealed low correlation (*r* ^2^ = 0.09) between passage timing at Bonneville Dam and tributary arrival timing for the inland lineage, which may suggest that these phenotypes may be independently controlled within this lineage, with restricted timing of passage at Bonneville Dam limited by low variation in the GREB1L gene region and tributary arrival timing more directly associated with SNPs in the intergenic region. Of 113 populations examined by Collins et al. (2020), the vast majority were from the inland lineage and very few had higher frequency of early than late haplotypes. Notably, populations in the Middle Fork Salmon River and upper Salmon River had relatively high frequency of early alleles from intergenic markers but only one, Chamberlain Creek, had a majority of early alleles (Table 1; reproduced from Collins et al., 2020; Willis et al., 2020; Willis et al., 2023).

**TABLE 1:** Frequency of early/late alleles for SNP at position Omy 11,618,027 (marker 3, “Omy_GREB1_05”) for coastal and inland lineages of Steelhead in the Columbia River.

Data set	Allele	Coastal	Intermediate	Inland
Steelhead returns over Bonneville Dam (Willis et al., 2021)				
		Allele frequencies		
	T (early)	82.7%	93.3%	4.6%
	G (late)	17.3%	6.7%	95.4%
Based on population‐level genotypes (Collins et al., 2020)				
		Allele frequencies		
	T (early)	38.8%	43.5%	9.0%
	G (late)	61.2%	56.5%	91.0%
Based on population‐level genome sequencing (Willis et al., 2023)				
		Allele frequencies		
	T (early)	0.494	0.769	0.123
	G (late)	0.506	0.231	0.877

*Note*: Populations at the crest of the Cascade Mountains are labeled as “intermediate” based on designations from Collins et al. (2020). Frequencies are shown for three different data sets based on analyses of individual genotypes from Bonneville Dam (Willis et al., 2020), population‐level genotypes (Collins et al., 2020), or population‐level genome sequencing (Willis et al., 2023).

**FIGURE 8:**
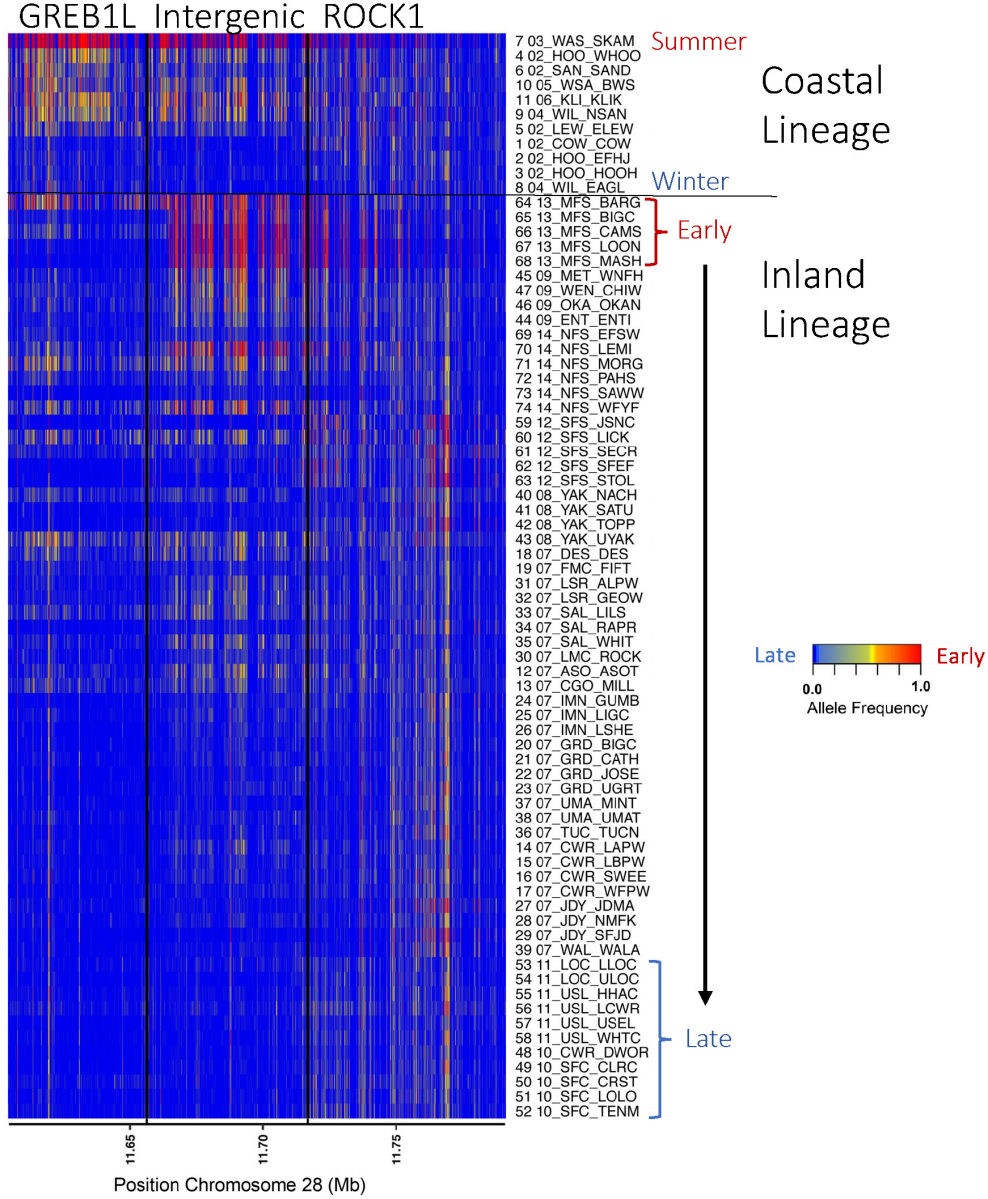
Allele frequencies for hundreds of SNPs spanning GREB1 through ROCK1 for 74 populations of Steelhead based on whole genome sequencing (Willis et al., 2023). Populations are sorted within each lineage by early at the top and late at the bottom. The coastal lineage includes the Skamania stock at the top that is nearly fixed for early alleles due to artificial selection. The legend shows the scale of frequency for populations ranging from early (red) to late (blue).

Given the complexity of genotype–phenotype associations for lineages of Steelhead within the Columbia River basin, more extensive whole genome resequencing was determined necessary to further examine variation from all SNPs in the GREB1L through ROCK1 region (Willis et al., 2023). A total of 74 populations of Steelhead (*n* = 4873) were individually barcoded and sequenced at low depth to estimate allele frequencies within each population to test for differences among groups. The coastal lineage included populations that represented summer versus winter migration timing and also included the Skamania strain that exhibits extreme early summer migration and acts as an effective reference collection for alleles associated with early migration. Differences in allele frequency between early and late collections of coastal lineage Steelhead indicated the breadth of association for this phenotype spanned approximately 112 kb from the GREB1L region through the intergenic region and into the 5′ end of ROCK1. In contrast, the inland lineage was represented by Steelhead which had a gradient of passage timing over Bonneville Dam, with the earliest returning fish from Middle Fork Salmon and the latest returning fish from populations in the Clearwater River (Hess, Ackerman, et al., 2016). This lineage generally had low variation in the GREB1L gene region compared with high variation through the intergenic region, and the breadth of association in the inland lineage was estimated to be only ~26 kb of the intergenic region of Omy28 (with exceptions in a few populations; Figure 8 reproduced from Willis et al., 2023). Examination of allele frequencies across populations suggested that SNPs in the intergenic region were the most informative for adult migration timing of inland lineage Steelhead of the Columbia River, and early alleles in this intergenic region were more common in regional populations such as the upper Columbia, Klickitat, lower Salmon, Middle Fork Salmon, and upper Salmon rivers (Willis et al., 2023). The loss of variation in the GREB1L region may be directly related to the constricted return timing of inland lineage steelhead, especially if this gene directly effects freshwater entry timing.

**FIGURE 9:**
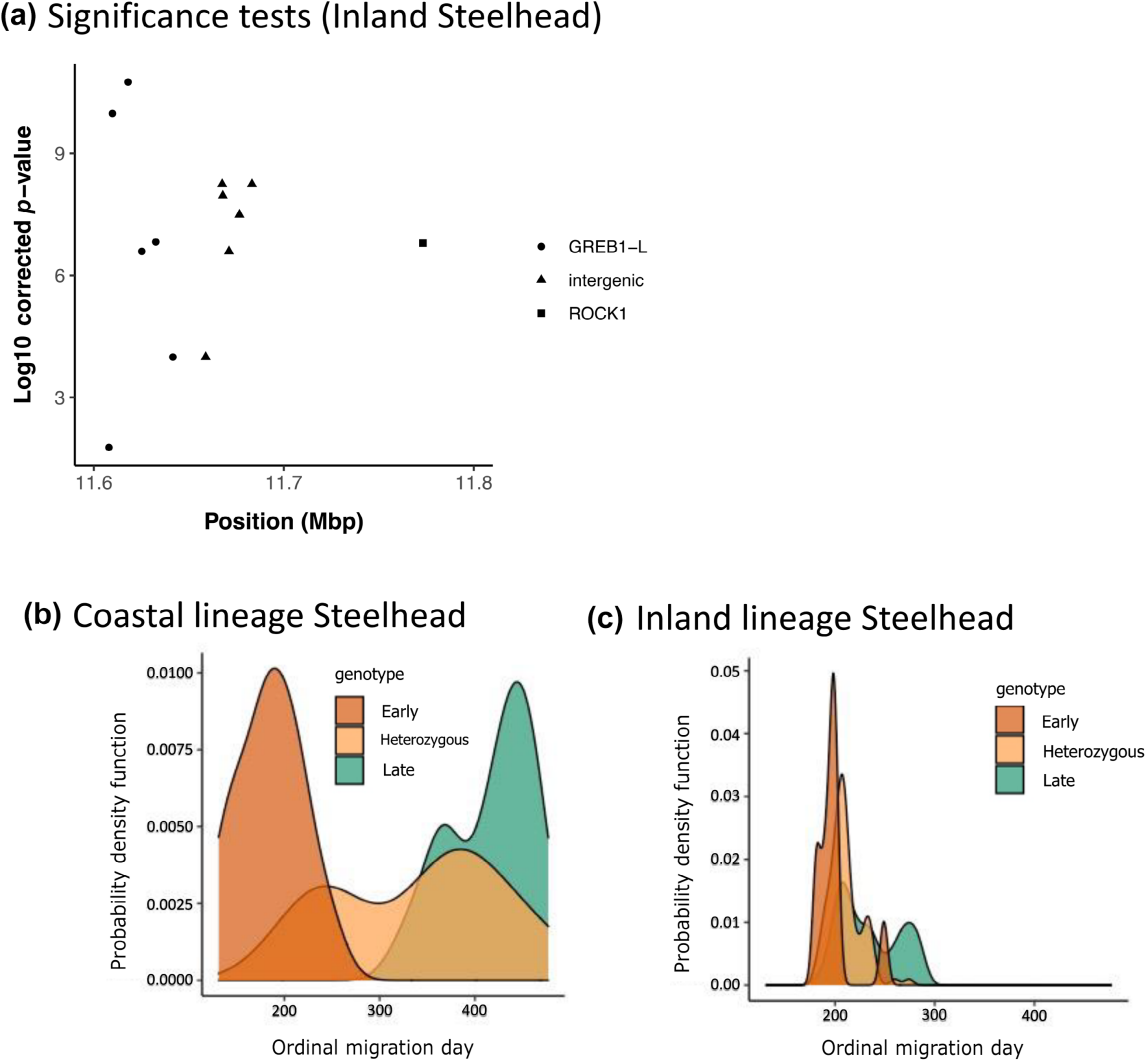
Re‐analyses of data from Willis et al. (2020) focused on inland Steelhead and patterns of association with Bonneville passage timing. (a) Significance of association for 13 markers from Omy28 with passage timing at Bonneville Dam for Inland Steelhead (marker 3 most statistically significant). (b) Relative genotype patterns for marker 3 for coastal Steelhead and passage timing at Bonneville Dam. (c) Genotype patterns for marker 3 from the GREB1L linkage block for Inland Steelhead passage timing at Bonneville Dam. Individual genotypes for homozygous early (brown), heterozygous (yellow), or homozygous late (green; Willis et al., 2020).

Based on results from population‐level whole‐genome resequencing, re‐analyses of data from Willis et al. (2020) focused on testing the association of 13 markers for Steelhead that were identified as inland lineage that overwintered in mainstem sites before returning to tributaries to spawn in the spring. Further analyses that separated inland lineage individuals from other Steelhead revealed that the most statistically significant association for passage timing over Bonneville Dam occurred for marker 3 in the GREB1L linkage block with heterozygotes generally exhibiting intermediate return timing (Figure 9a). The intergenic markers from the second linkage block were also associated with passage timing over Bonneville Dam, but heterozygous fish showed partially dominant phenotypic effects for early alleles (Willis et al., 2021). The effect size on Bonneville passage timing was relatively low (2.5%) for inland Steelhead in these analyses, but patterns of migration for passage timing over Bonneville Dam were evident and enabled comparison between lineages at a more detailed level than previous analyses (Figure 9b,c). These newest results validate that the GREB1L/intergenic region of Omy28 has phenotypic effects on migration timing phenotypes for inland lineage Steelhead, but the mechanisms of GREB1L expression on adult migration phenotypes remain unknown.

Following the discovery of association of the GREB1L/intergenic region of Omy28 with adult migration timing in Steelhead, several studies have applied markers from this region to investigate migration phenotypes of Steelhead in specific study systems in the Columbia River basin and in other regions of the species' range. Studies within specific systems include evaluation of the frequency of alleles associated with summer versus winter migration as related to conservation efforts in the South Santiam River, Oregon (Weigel et al., 2019) and the Klickitat River, WA (Collins et al., 2023). Both studies provided evidence that interbreeding occurs between early and late migrating steelhead when opportunities exist, either naturally (Collins et al., 2023) or artificially (Weigel et al., 2019). However, the frequency of heterozygous adults was lower than expected and suggests selection may act against intermediate run‐timing and favor Steelhead with run‐timing that is adapted to local stream systems. Alternatively, Wahlund effect may contribute to heterozygote deficiency due to limited interbreeding between summer‐ and winter‐run steelhead in this system. Another study of native Redband Trout populations that are now restricted from anadromy provided evidence that resident populations harbor variation for both early and late alleles at Omy28, but it is unknown if this variation affects phenotypes within freshwater populations such as timing of arrival for spawning or mating (Andrews et al., 2023). Similarly, studies of the GREB1L region of Omy28 in populations of *O. mykiss* provide support for locally adapted migration timing based on the relative frequency of associated alleles (Fraik et al., 2021; Kannry et al., 2020).

## 3 CHINOOK SALMON—SYNTHESIS OF PATTERNS OF GENOMIC ASSOCIATION WITH MIGRATION TIMING

Chinook Salmon of the Columbia River exhibit high levels of genetic divergence between coastal and interior populations, similar to Steelhead and several other fish species in this ecosystem (e.g., Quinn, 2018). Geological conditions over evolutionary time have led to three extant lineages of Chinook Salmon (Waples et al., 2008), which include the coastal lineage (lower Columbia River, west of the Cascade Mountains) and two interior lineages to the east known as interior ocean type and interior stream type (e.g., Narum et al., 2010). Notably, the interior stream type is most genetically divergent from the other two lineages (Hecht et al., 2015; Moran et al., 2013; Narum et al., 2010; Waples et al., 2004) to the extent it could be considered a distinct sub‐species (Waples et al., 2004). Migration distance is one of the most substantial differences between coastal and interior lineages, with evidence supporting local adaptation through environmental drivers of selection within and among populations (e.g., Hecht et al., 2015). Several populations comprise each of these major lineages with metapopulation structure that has led to designation of eight distinct population segments (DPS) of Chinook Salmon (Myers et al., 1998; Waples et al., 2001). Of the 8 DPS/ESU for Chinook Salmon in the Columbia River, the ESA listings include 1 that is Endangered (Upper Columbia River Spring), 4 as Threatened (Lower Columbia River, Snake River Spring/Summer, Snake River Fall, Upper Willamette River), and 3 that are not listed (Middle Columbia River, Deschutes River Summer/Fall, Upper Columbia Summer/Fall).

**FIGURE 10:**
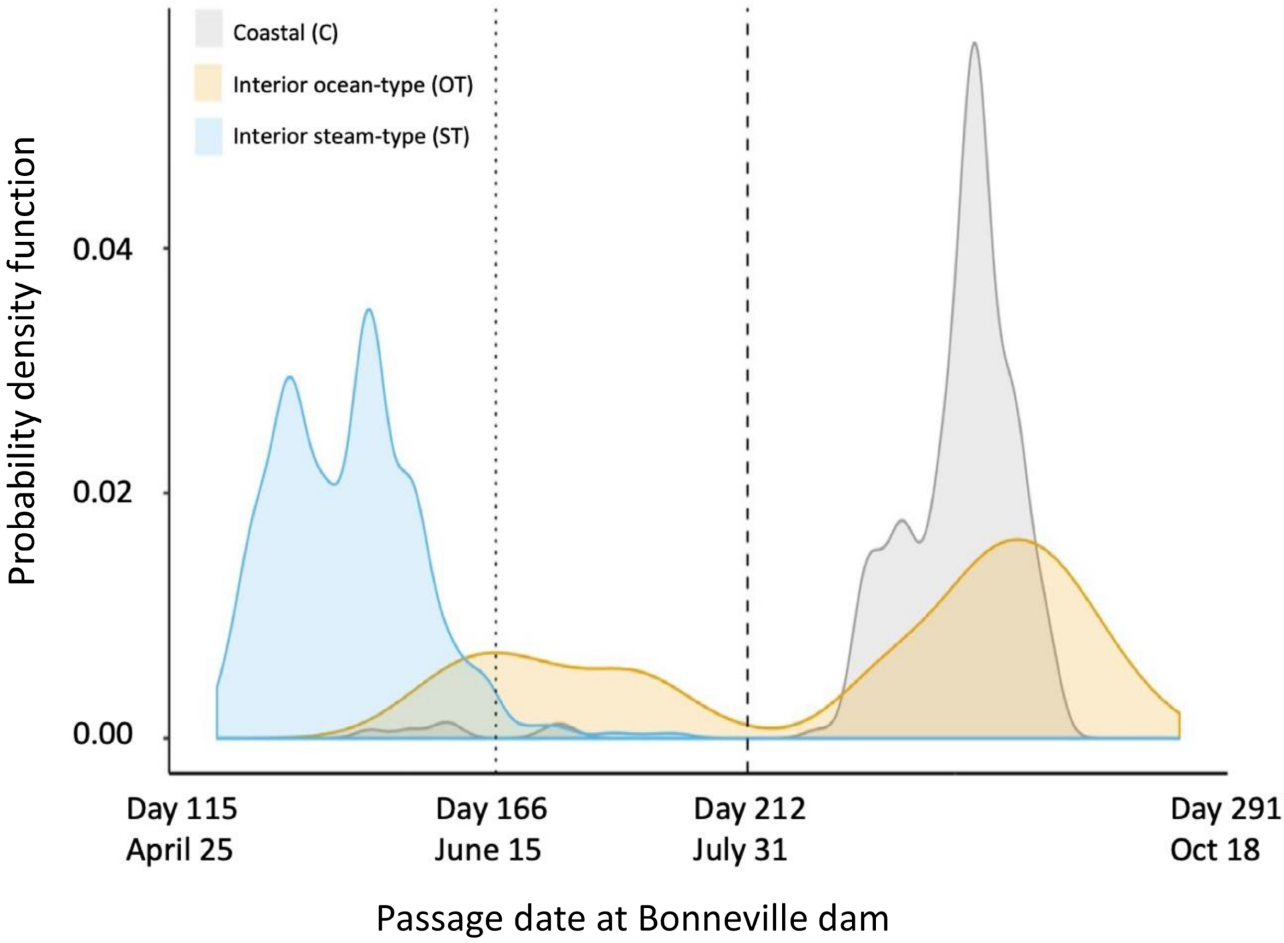
Chinook Salmon passage timing over Bonneville Dam is designated for the three major genetic lineages that occur in the Columbia River (Willis et al., 2021). The probability density function for the date at which each Columbia River Basin Chinook Salmon was tagged crossing Bonneville Dam between April and October, for each lineage (gray, Coastal; orange, interior ocean type; blue, interior stream type). The probability density function is akin to the proportion of fish in a lineage that passed on each day. For each color, the area under the curve sums to 1, but curves are not proportional to each other in counts; the sample size representative of each curve is indicated. Vertical dotted and dashed lines represent divisions between the spring, summer, and fall management periods, ordinal, and calendar dates for which are noted, along with the first and last days a Chinook Salmon in this data set migrated.

Adult migration timing differs substantially among lineages of Chinook Salmon, with coastal populations comprised of spring and fall migrating adults that are typically found in sympatry throughout the PNW from California to British Columbia (e.g., Myers et al., 1998). In contrast, Chinook Salmon from the two interior lineages of the Columbia River return with distinct run‐timing due to the long migration distance individuals from these populations must travel to return to spawning grounds (Quinn et al., 2016). Populations of interior ocean‐type lineage only return in summer through fall months with no spring run, and spawning occurs in fall (Hess et al., 2014; Keefer et al., 2004; Quinn et al., 2016). Populations of interior stream‐type lineage have the most restricted range of return timing from spring through summer months with no fall run, and spawning occurring in late summer (Hess et al., 2014; Keefer et al., 2004). Thus, the two interior lineages of Chinook Salmon have distinct return timing and less separation in peak migration timing of early and late modes relative to the typical migration timing of adults in coastal populations. Specifically, early and late migrating fish are represented within each lineage (Figure 10; adapted from Willis et al., 2021) with coastal lineage considered spring (early) or fall (late), and interior ocean type considered summer (early) or fall (late), and interior stream‐type considered spring (early) or summer (late).

Many genetic studies of Chinook Salmon populations from the PNW have occurred over the past decades with several types of putatively neutral markers, such as allozymes (e.g., Waples et al., 2004), mtDNA (e.g., Martin et al., 2010), microsatellites (e.g., Seeb et al., 2007), and modest numbers of SNPs (Narum et al. 2008). All studies, regardless of marker type, provided support for at least three major lineages within the Columbia River basin and distinct populations within each lineage that reflect limited gene flow due to philopatry to natal rivers. However, there is greater genetic similarity between migratory types within populations than between populations of each lineage (reviewed by Waples et al., 2022). For several decades, Chinook Salmon have been managed by the calendar date in which they are found in the mainstem Columbia River and have been classified into Spring (January 1–June 15), Summer (June 16–August 31), and Fall (August 1–December 31) runs (Myers et al., 1998; Keefer et al., 2004). These designations are part of U.S. v OR Management Agreement policy decisions to meet hydropower mitigation that generally balance protections of ESA listed stocks (e.g., natural origin Snake River Spring Chinook Salmon) while allowing harvest on non‐ESA listed stocks (e.g., upper Columbia Summer Chinook Salmon). Although biological information on the genetic lineages of Chinook Salmon was taken into account as part of these policy decisions, managers recognize that these management periods do not provide a perfect means to separate genetic lineages and there is some degree of overlap of lineages across periods (Hess et al., 2014; Willis et al., 2021). For example, late migrating interior stream‐type Chinook Salmon are primarily found in the Columbia River mainstem during the Spring Period but a relatively small portion overlaps the Summer Period (Figure 10). Similarly, interior ocean‐type individuals from the upper Columbia River summer‐run population overlap the Spring Period, although most will occur in the mainstem during the Summer Period (Hess et al., 2014; Willis et al., 2021).

Recent studies have implemented reduced representation sequencing to investigate the genetic basis for adult migration timing in Chinook Salmon populations with initial genome scan studies covering <1% of the genome (Brieuc et al., 2015; Prince et al., 2017). The study by Brieuc et al. (2015) considered populations across multiple lineages with various run‐timing but analyzed all populations for outliers regardless of lineage which led to unclear signals of significance across multiple linkage groups and the GREB1L gene region was not identified. Another study by Prince et al. (2017) focused on spring versus fall run‐timing in coastal populations of Chinook Salmon from the PNW and identified markers near the GREB1L gene on Ots28 as statistically associated with adult migration timing. While this candidate region from GREB1L was intriguing, especially given the concordant finding in Steelhead from Prince et al. (2017), further research was necessary to test for variation in run‐timing in other lineages such as those in the Columbia River with high marker density to pinpoint patterns of association with adult migration timing.

**FIGURE 11:**
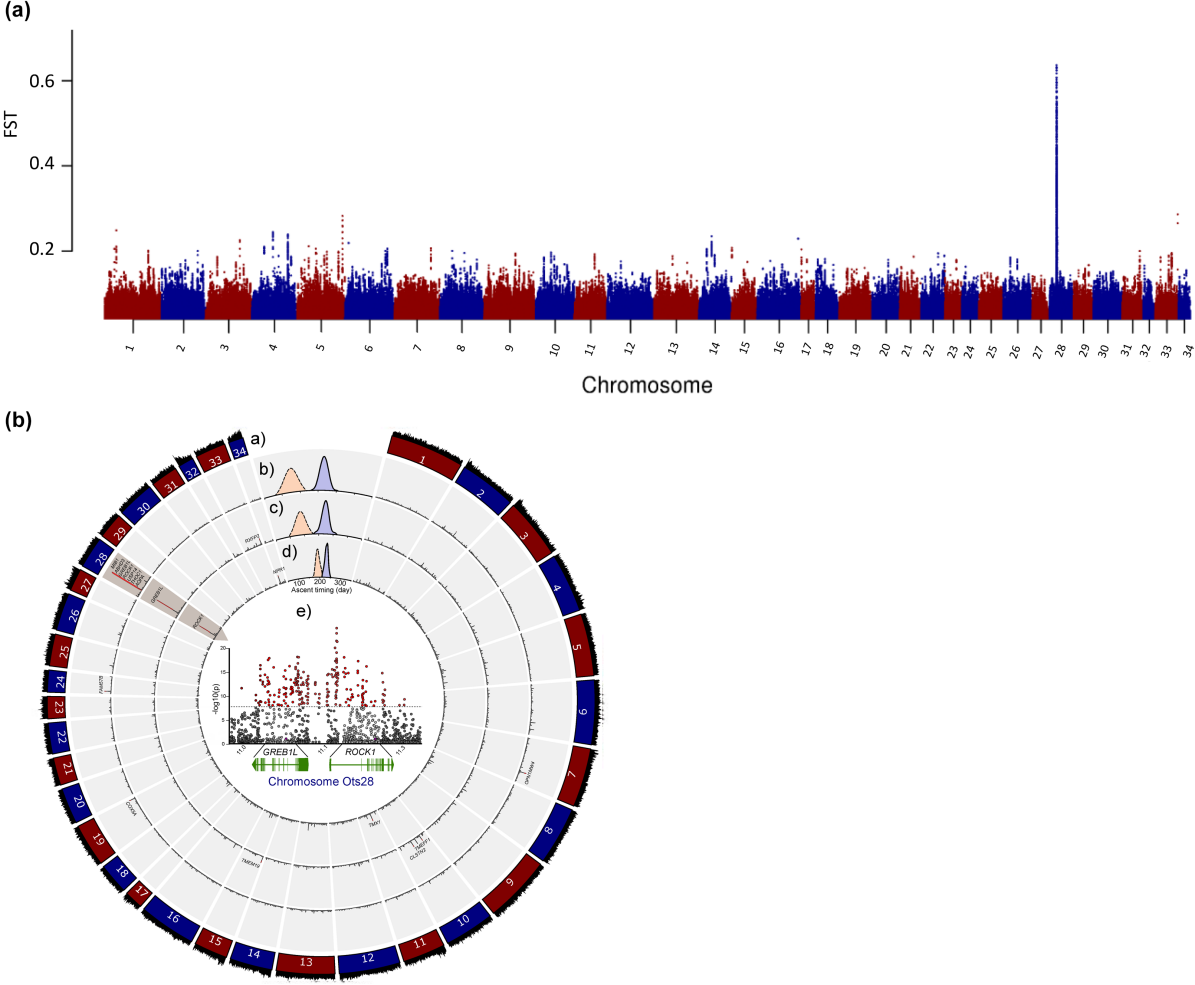
Genomic basis for adult migration timing in Chinook Salmon lineages from the Columbia River. (a) Manhattan plot of genetic divergence between early and late migrating populations of Chinook Salmon across the *O. tshawytscha* genome, with the most substantial peak of FST at Chromosome 28 (Ots28; Narum et al., 2018). (b) Concentric rings of Manhattan plots of b.a) sequence coverage, and for early (orange peak) or late (blue peak) groups for each major lineage of Chinook Salmon b.b) coastal, b.c) interior b.c ocean type, b.d) interior stream type, with b.e) the statistically significant SNPs (CMH tests) within the region for GREB1L through ROCK1 shown in the center of the circle (Narum et al., 2018). Annotation included for the 203 kb region on Ots28 between 11.022 and 11.225 Mb (GREB1 L, ROCK1, and intergenic regions from assembly GCA_002831465.1). Details for additional genes labeled in the Manhattan plots are available from Narum et al. (2018).

**FIGURE 12:**
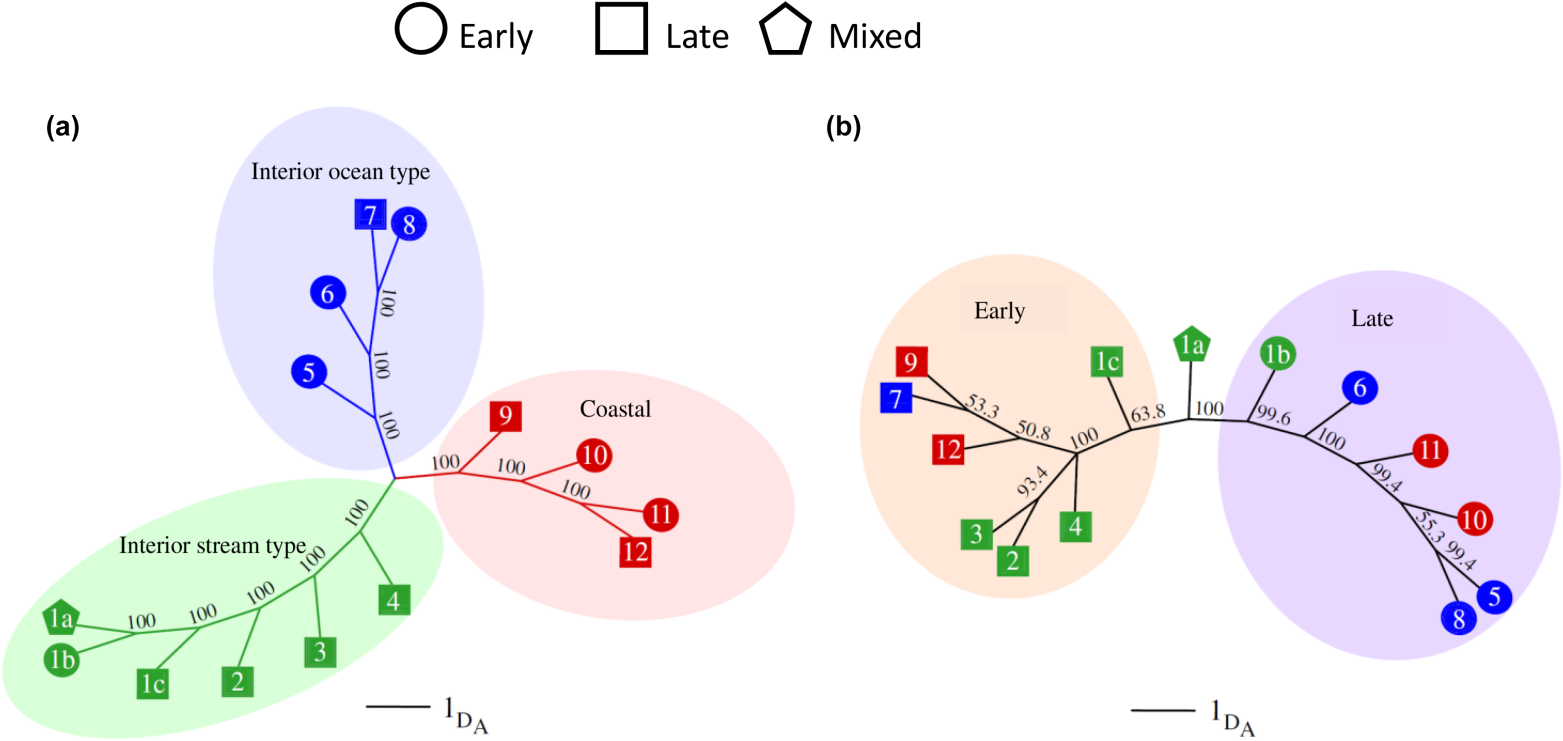
Neighbor‐joining trees of Nei's genetic distance representing distinct genetic relationships among populations of Chinook Salmon (Narum et al., 2018) based on either (a) putatively neutral SNPs; patterns support known genetic structure split by lineage (coastal = red circles, interior ocean type = blue circles, interior stream type = green circles). (b) SNPs in GREB1L/ROCK1 region; patterns support clustering by adult migration timing regardless of lineage (orange shaded oval for early migration, purple shaded oval for late migration). Collection numbers correspond to site list in supplementary material from Narum et al. (2018; table S1, figure 1). “Mixed” indicates adult migration timing phenotypes that were combined in one collection.

A subsequent study implemented whole genome resequencing between replicate collections for each of the three major lineages of Chinook Salmon in the Columbia River to test for genomic differences in run‐timing within each lineage (Narum et al., 2018). A de novo reference genome was also assembled with intensive sequencing of one male fish from the interior stream‐type lineage (Johnson Creek) to enable mapping of resequencing data and was the first chromosome‐level genome assembly released for Chinook Salmon (Narum et al., 2018; male assembly of 2.4 Gb; NCBI accession GCA_002831465.1), with other genome assemblies for this species released shortly thereafter for the coastal lineage (Christensen et al., 2018; NCBI accession GCA_002872995.1 female assembly of 2.4 Gb; and NCBI accession GCA_018296145.1 male assembly of 2.3 Gb). Narum et al. (2018) applied a Pool‐Seq approach (e.g., Schlötterer et al., 2014) to sequence 67.2% of the genome at 33× coverage on average for collections that represented distinct run‐timing within each lineage. Genome scans for differences in allele frequency across 19 million SNPs revealed multiple regions of significance, but the strongest and most consistent signal for differences in run‐timing for each lineage occurred along a span from GREB1L through an adjacent gene, ROCK1, on Ots28 (Figure 11, reproduced from Narum et al., 2018). The most statistically significant SNPs within this region varied by lineage, but the strongest differences in allele frequency consistently occurred in the intergenic region nearest ROCK1 and provided distinct relationships related to adult migration timing rather than neutral processes such as gene flow (Figure 12, reproduced from Narum et al., 2018). This study identified that the genomic extent of run‐timing for Chinook Salmon was largely associated with variation within this region of Ots28, providing more extensive support for this candidate region.

However, further studies were necessary to validate patterns of association and estimate the effect size of this candidate region with adult migration timing in Chinook Salmon. The most statistically significant SNP markers from Narum et al. (2018) were selected for development into amplicon sequencing assays to enable validation testing with a large number of independent samples and more extensive phenotypes of passage timing at Bonneville Dam, tributary arrival timing, and arrival timing for spawning. A total of 28 SNP markers were successfully incorporated into GT‐seq panels for this species, with 5 SNPs in the GREB1L region, 11 SNPs in the intergenic region, and 12 SNPs in the ROCK1 region after 5 were dropped from initial panels due to inconsistent amplification (Koch & Narum, 2020; Willis et al., 2021). Primers and probes for these 28 markers, and the key for early versus late alleles in Chinook Salmon, are provided in Table S2 (reproduced from Koch & Narum, 2020; Willis et al., 2021). Two follow‐up studies provided validation for patterns of association with adult migration timing in Chinook Salmon (Koch & Narum, 2020; Willis et al., 2021), with both studies focused on the three lineages from the Columbia River basin.

**FIGURE 13:**
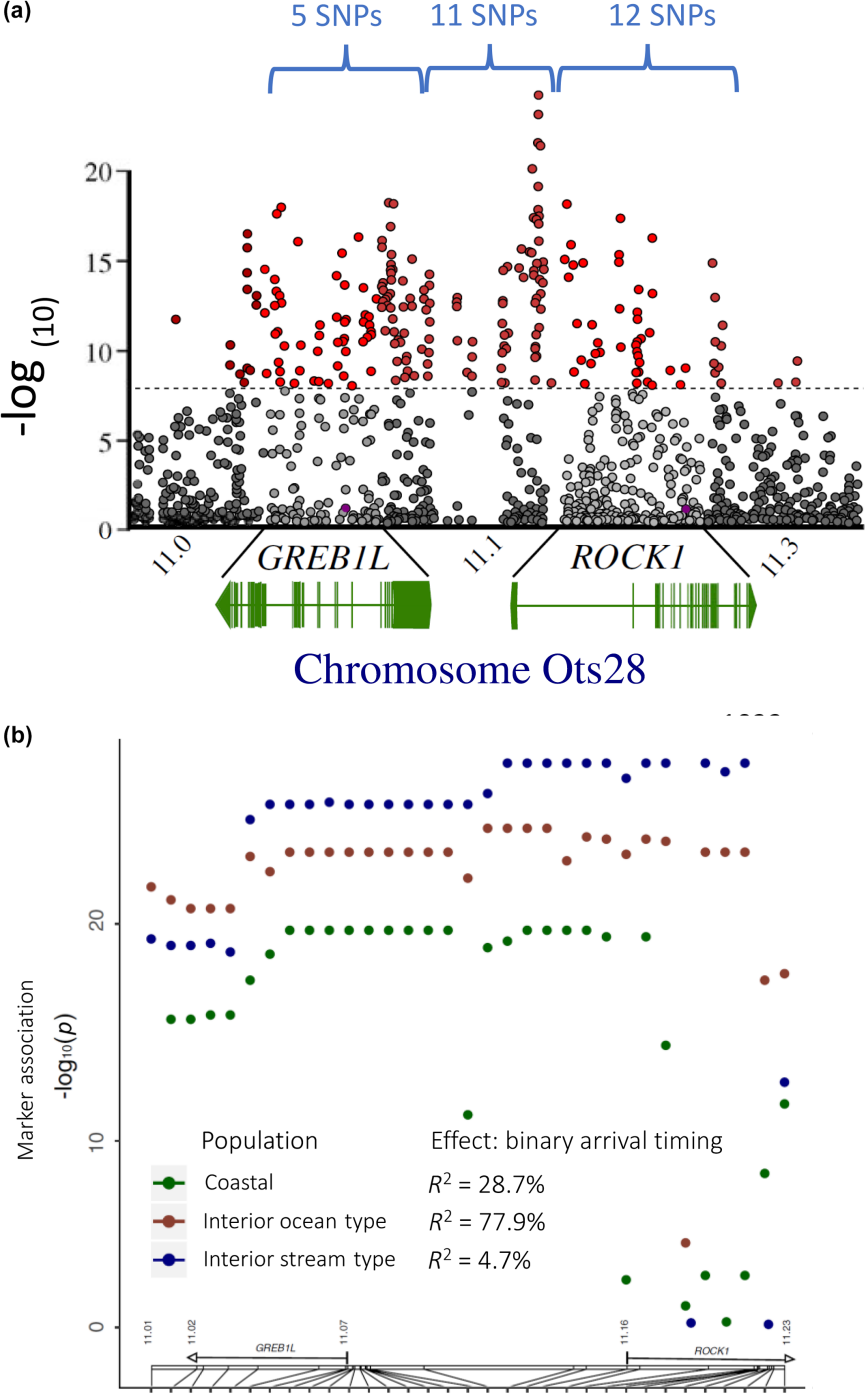
Development of candidate SNPs for adult migration timing for genotyping in GT‐seq panels for Chinook Salmon. (a) A total of 28 of the most statistically significant SNPs were successfully designed for high throughput genotyping assays with 5 SNPs from the GREB1L region, 11 from intergenic, and 12 from ROCK1 (Narum et al., 2018). (b) Patterns of association for SNP markers and arrival timing for spawning for one population from each lineage of Chinook Salmon (Koch & Narum, 2020). The genome position of markers on chromosome 28 is depicted in the gene diagrams above the x‐axis. The y‐axis represents −log10 (FDR‐corrected p‐value; Koch & Narum, 2020).

The first validation study included early vs. late migrating collections within a population of each lineage, with all samples genotyped at the candidate markers from GREB1L/ROCK1 (Koch & Narum, 2020). Association tests confirmed most candidate markers on Ots28 were statistically significantly associated with binary arrival timing for spawning and the strongest association for all three lineages was consistently observed for SNPs within or near the 5′ end of the ROCK1 gene (Figure 13 reproduced from Narum et al., 2018; Koch & Narum, 2020). However, patterns of linkage differed, with a single linkage block observed for these markers in the coastal and interior ocean‐type lineage, but two distinct linkage blocks for the interior stream‐type lineage (one block for markers in GREB1L and upstream, second block for markers in ROCK1 and markers upstream). Further, the effect size differed by lineage, with the largest effect (78% of variation explained) observed between fish arriving for spawning in summer (early) vs. fall (late) in the interior ocean‐type lineage, followed by an effect size of 29% between fish arriving for spawning in spring (early) versus fall (late) in the coastal lineage, and a modest effect size of 5% for fish arriving for spawning in early versus late‐arriving fish in the interior stream‐type lineage (Koch & Narum, 2020). Differences in effect size among lineages could be due to total variance within each lineage (Gene–Environment interaction; GxE), or sampling effects among lineages that could have influenced results such as estimation of phenotypes of arrival timing for spawning. Tests of heterozygous fish indicated potential dominant effects of early alleles from ROCK1 on arrival timing for spawning, but also lower fitness for fish with early alleles in the interior stream‐type lineage (Koch & Narum, 2020). Thus, this first validation study provided validation for association and effect size of the GREB1L/ROCK1 region with the adult migration phenotype of arrival timing for spawning, but for a limited number of populations.

**FIGURE 14:**
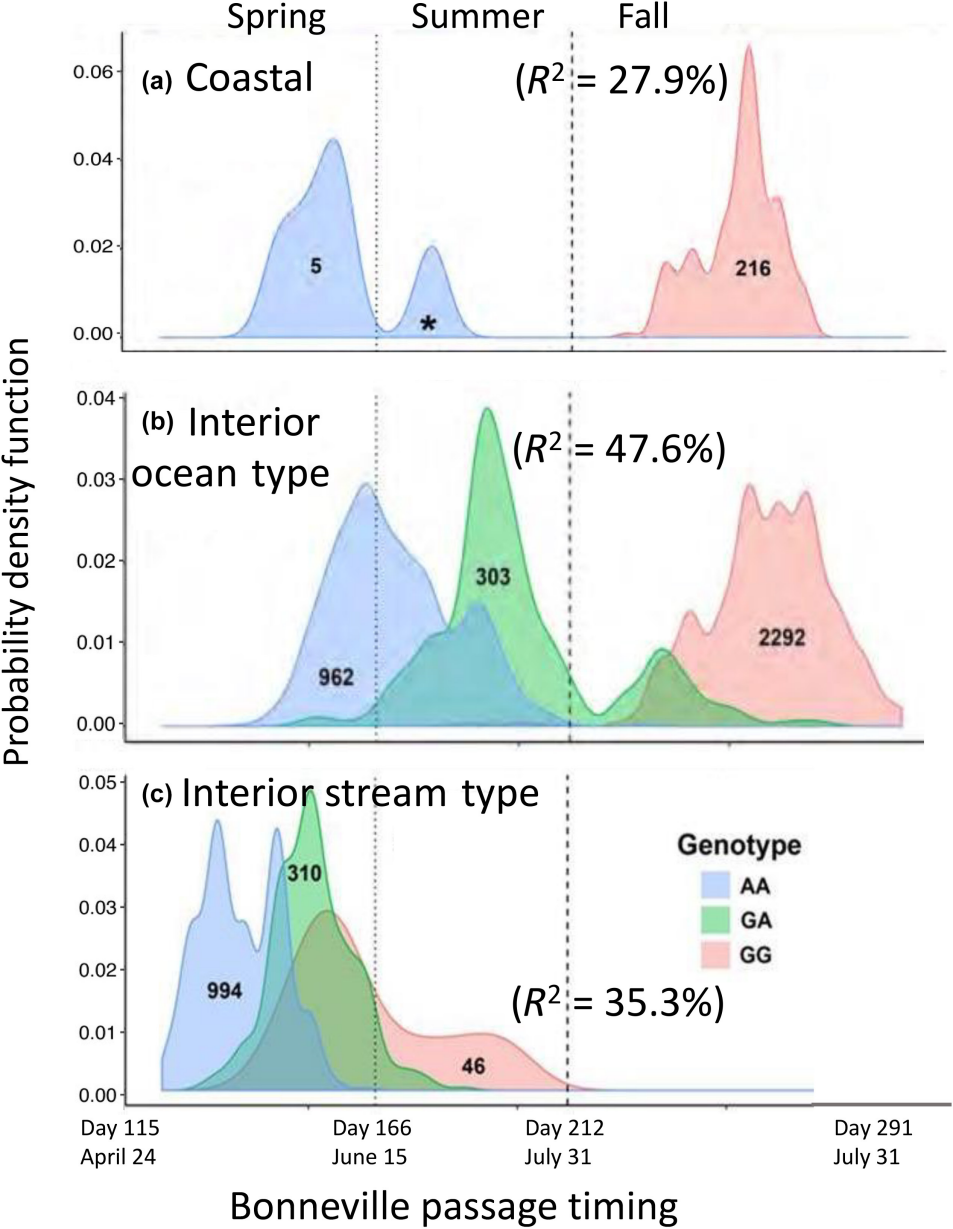
Genotype to phenotype association for each lineage of Chinook Salmon and passage timing at Bonneville Dam during spring, summer, and fall management periods based on individual genotypes for homozygous early (blue, AA), heterozygous (green, GA), or homozygous late (red, GG) at a single representative marker (Ots28_11143508) from the most strongly associated linkage group, Willis et al. (2021). (a) Coastal lineage (effect size 27.9%; the passage of a single heterozygote is indicated with an asterisk), (b) interior ocean type (effect size = 47.6%), (c) interior stream type (effect size 35.3%). For each color on each panel, the area under the curve sums to 1, but curves are not proportional to each other in counts; the sample size for each curve is indicated. Vertical dotted and dashed lines represent divisions between the spring, summer, and fall management periods, ordinal, and calendar dates for which are noted, along with the first and last days a Chinook Salmon in the data set migrated.

The second validation study incorporated samples representing a broader set of populations and phenotypes (*n* = 5149) for adult Chinook Salmon returning to the Columbia River, which were genotyped with the 28 SNPs from GREB1L/ROCK1 along with hundreds of neutral markers to distinguish fish by lineage (Willis et al., 2021). Phenotypes for individuals were based on collection date at Bonneville Dam (passage timing at Bonneville Dam by ordinal day of the year), and PIT‐tagged fish were tracked at arrays within tributaries to estimate tributary arrival timing. Results from this study once again identified markers from the intergenic region nearest ROCK1 as the most statistically significant in each lineage, but with different effect sizes. Genotypes explained between 28% and 48% of variation in passage timing at Bonneville Dam, with weaker but substantial effects for tributary arrival timing (10%–40% of variation). The effect size on passage timing at Bonneville Dam differed by lineage with a larger effect once again observed in the interior ocean‐type lineage (47.6% of variation) than in the coastal lineage (27.9% of variation). However, this study revealed a substantial effect size (35.3%) for passage timing at Bonneville Dam (ordinal day) in the interior stream‐type lineage (Figure 14 reproduced from Willis et al., 2021), which was not evident in the previous study that only included binary arrival phenotypes (Koch & Narum, 2020). This finding for interior stream‐type fish was one of the most substantial results of Willis et al. (2021) since it was the first to confirm the strong association and effect size of candidate markers with the narrow range of adult migration timing in this lineage.

**FIGURE 15:**
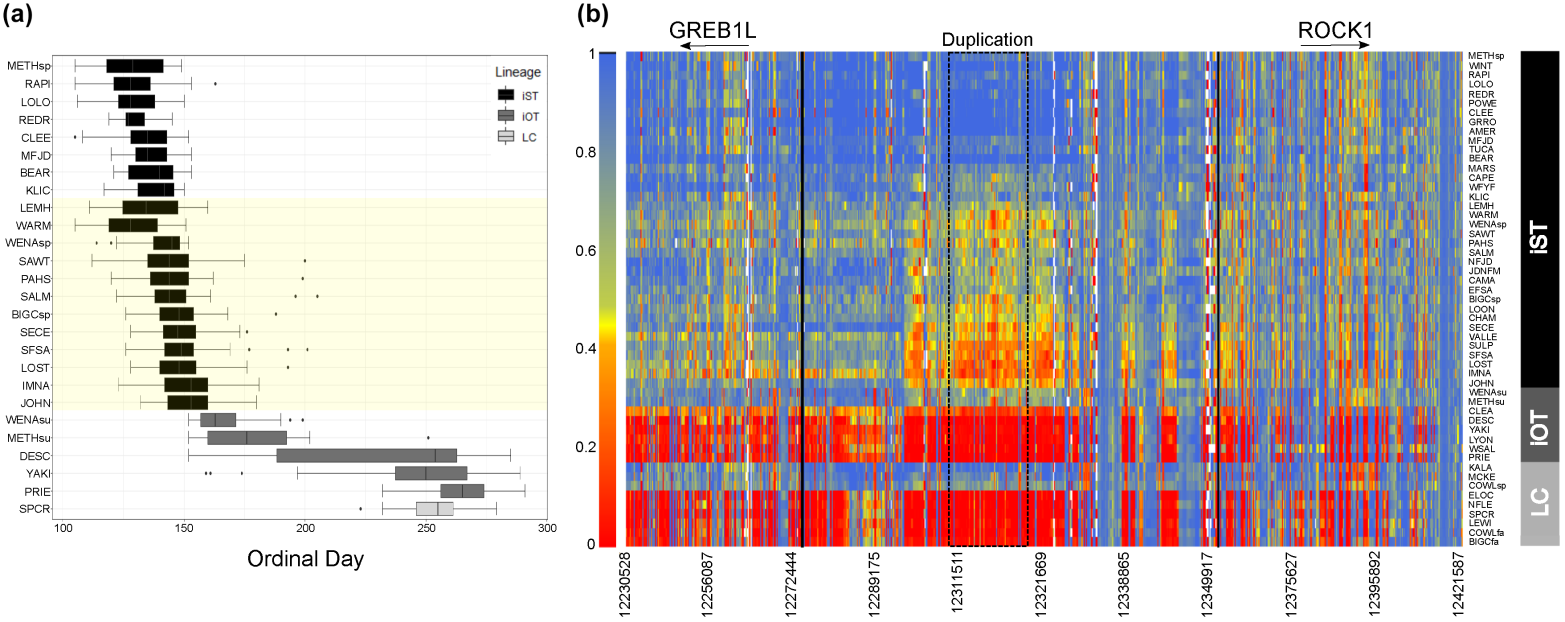
Allele frequencies for hundreds of SNPs spanning GREB1 through ROCK1 for 53 populations of Chinook Salmon based on whole genome sequencing (Horn & Narum, 2023). (a) Populations are sorted within each lineage by adult migration timing with early at the top and late at the bottom. Lineages include interior stream type (iST), interior ocean type (iOT), and coastal (Lower Columbia/LC). The yellow‐shaded box corresponds to populations in the iST lineage that exhibited heterozygosity for the duplicated block within the intergenic region as noted by Horn and Narum (2023). (b) Allele frequencies for populations within each lineage of Chinook Salmon with early allele frequencies in blue and late allele frequencies in red. The duplicated region found by Thompson et al. (2020) is outlined by a dashed box. For a list of population abbreviations, see table 1 in Horn and Narum (2023).

**FIGURE 16:**
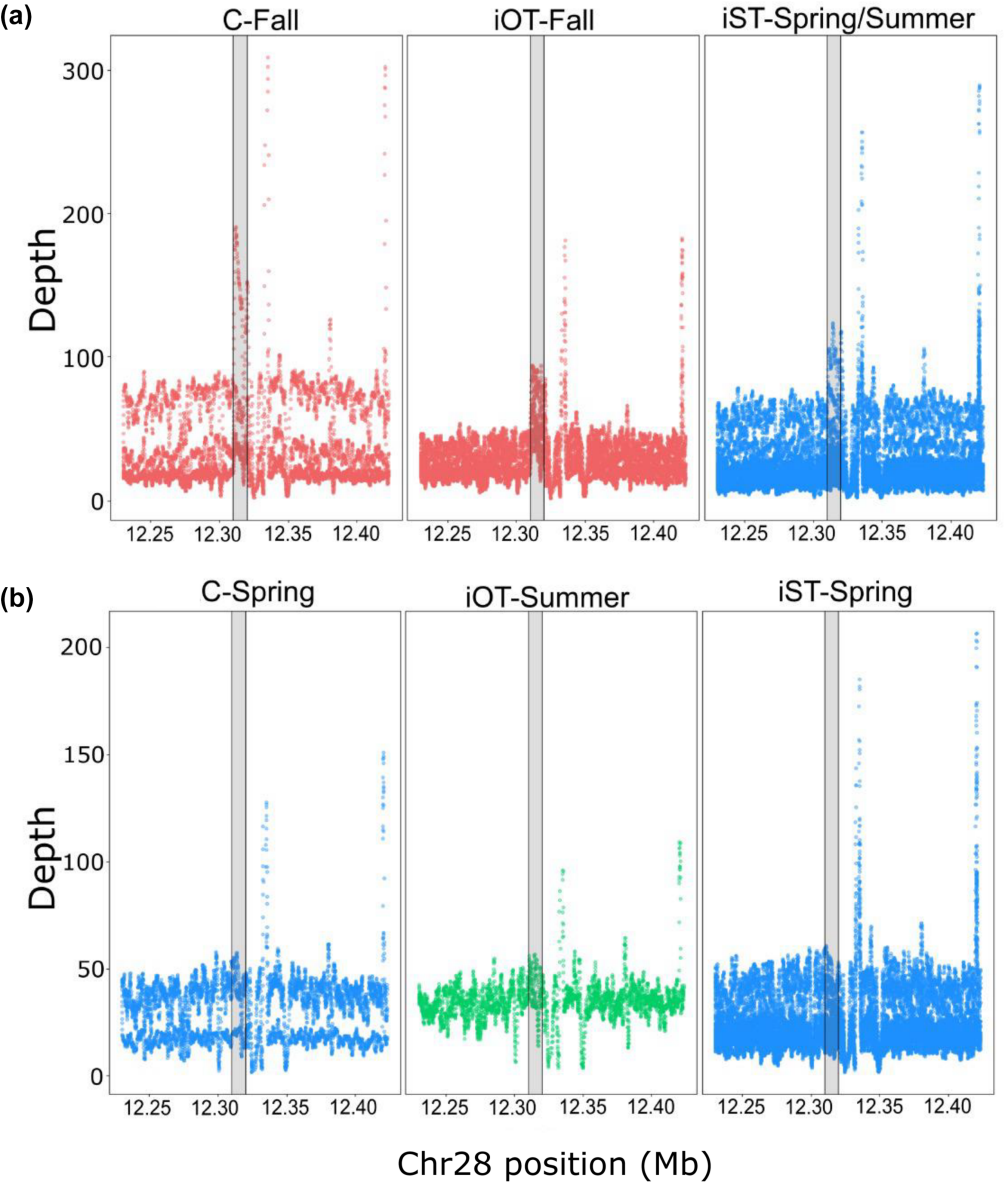
Contrast in read depth for the duplication in Ots28 intergenic region sorted by panels for each lineage of Chinook Salmon: left (Coastal = C), middle (interior ocean type = iOT), and right (interior stream type = iST) panels (Horn & Narum, 2023). (a) Late migrating populations of each lineage and (b) early phenotypes of each lineage. The gray shaded box represents the first duplicated region identified by Thompson et al. (2020) which was associated with adult migration phenotypes, while the two additional peaks represent other duplications that were not associated. For the coastal lineage, populations are split by spring (blue) versus fall (red) migration timing. In the interior ocean‐type lineage (iOT), populations are split by summer (green) versus fall migration timing. The iST populations are split between those populations displaying variation in copies for the duplicated block within the intergenic region between GREB1L and ROCK1 (iST‐Spring/Summer) and those without duplication (iST‐Spring).

Despite several intriguing results from validation studies, more thorough characterization of genomic variation in the GREB1L/ROCK1 region of Ots28 was necessary at the population level. This was further apparent after duplication was noted in this region of Ots28 in a study of coastal lineage Chinook Salmon from populations in California and Oregon (Thompson et al., 2020) that indicated structural variation may reduce recombination in this region and copy number may be related with early vs. late migration timing. A large whole genome resequencing study (Horn & Narum, 2023) of Chinook Salmon populations from the Columbia River enabled population‐level allele frequencies across the entire GREB1L/ROCK1 region and the ability to test for copy number effects on adult migration timing. A total of 53 populations were individually barcoded (n = 3566) and sequenced at low depth to estimate allele frequencies for ~13 million SNPs (Horn & Narum, 2023). The coastal lineage included populations that represented spring (early) versus fall (late) run‐timing, the interior ocean‐type lineage included populations of summer (early) and fall (late) run‐timing, while the interior stream‐type lineage included populations with a gradient of passage timing at Bonneville Dam ranging from spring (early) through summer (late). Differences in allele frequency between early and late collections indicated the breadth of association spanned approximately 200 Kb from the GREB1L region through intergenic and into the 5′ end of ROCK1 for both the coastal and interior ocean‐type lineages. In contrast, the interior stream‐type lineage was represented by Chinook Salmon populations that had a gradient of passage timing over Bonneville Dam, with the earliest returning fish from the upper Columbia River and Rapid River, and the latest returning fish from populations in the Salmon River (Hess et al., 2014). The interior stream‐type lineage generally had weaker differences in allele frequency within the GREB1L gene region compared with high differences in allele frequency in SNPs through the intergenic region, and the breadth of association in the interior stream‐type lineage was estimated to be only ~26 kb of the intergenic region of Ots28 (Figure 15, reproduced from Horn & Narum, 2023). Examination of allele frequencies across populations suggested that SNPs within the intergenic region were the most informative for adult migration timing in Chinook Salmon lineages of the Columbia River. Across all populations, one of the most diagnostic SNPs in this intergenic region (position 12,299,996 on Ots28) provided an indication of the frequency of early alleles throughout the Columbia River basin. Each lineage had a frequency of early alleles that ranged from modest in the coastal lineage (34%) and the interior ocean‐type lineage (31%) to high (85%) in the interior stream‐type lineage (Table 2, based on allele frequencies from Willis et al., 2021 and Horn et al. 2023). Further, the duplication noted by Thompson et al. (2020) was found to exhibit higher copy numbers in late‐returning fish from each lineage relative to early returning fish (Figure 16, reproduced from Horn & Narum, 2023), suggesting that structural variation in the form of this duplication within the intergenic region between GREB1L and ROCK1 may be a driver of phenotypic differences (Horn & Narum, 2023). These results validate that the GREB1L/intergenic region of Ots28 has phenotypic effects on adult migration timing phenotypes for all lineages of Chinook Salmon in the Columbia River, but the mechanisms of expression on any specific migration phenotypes remain unknown.

**TABLE 2:** Frequency of early/late alleles for SNP at position Ots 12,299,996 (marker 15, “Ots28_11095755”) for each of three lineages of Chinook Salmon (coastal, interior ocean type = “iOT,” interior stream type = “iST”), based on analyses of individual genotypes from Bonneville Dam (Willis et al., 2021) or population‐level genome sequencing (Horn & Narum, 2023).

Dataset	Allele	Lineage of Chinook Salmon		
		Coastal	iOT	iST
Chinook Salmon returns over Bonneville Dam (Willis et al., 2021)				
		Allele frequencies		
	T (early)	2.5%	31.7%	84.9%
	A (late)	97.5%	68.3%	15.1%
Based on estimates within population samples (Horn & Narum, 2023)				
		Allele frequencies		
	T (early)	33.8%	20.5%	74.3%
	A (late)	66.2%	79.5%	25.7%

## 4 FURTHER RESEARCH QUESTIONS FOR STEELHEAD AND CHINOOK SALMON

While much research has been completed in recent years that elucidates GREB1L/ROCK1 as a major effect region for adult migration timing in Steelhead and Chinook Salmon, there are many remaining questions that require further study. A list of research to address uncertainties in this regard is noted in the review by Waples et al. (2022), but further guidance is provided here to compel future studies.

- *Are there fitness effects of early* vs. *late migration?*
  While numerous studies have found that fitness differs for in Steelhead and Chinook with different adult migration timings (reviewed in Koch & Narum, 2021), direct estimates of the association between alleles across GREB1L/ROCK1 and fitness have been limited. Koch and Narum (2020) found a statistical association between male fitness and one of the SNPs within ROCK1, but this effect was not found in females. Further, the relationship between fitness and the GREB1L/ROCK1 region was only measured in a single interior stream‐type population in this study. The maintenance of variation at large‐effect genomic regions is thought to be maintained by variation in fitness effects across individuals, environments, or time (reviewed in Waples et al., 2022) as would be expected with strong balancing selection. Thus, it is possible that frequency‐dependent selection and intralocus sexual conflict, whereby there are distinct fitness optimums in migration timing for each sex, potentially contribute to the maintenance of this large‐effect polymorphism at GREB1L/ROCK1. Future studies are needed to directly measure the association between fitness and SNPs spanning the GREB1L/ROCK1 region across different Chinook Salmon and Steelhead populations, while also controlling for other life history factors, such as sex, size, origin, and age at maturity. Evaluation of fitness for individuals that have heterozygous genotypes in GREB1L/ROCK1 is a particular area of future study since fish with intermediate migration timing may experience distinct selection pressure.
- *Is there phenotypic variation in timing of various reproductive traits in addition to adult migration timing?*
  Populations of Chinook Salmon and Steelhead that make long‐distance migrations during their return to natal spawning areas exhibit highly complex phenotypes for adult migration timing beyond freshwater entry that has been the primary focus in coastal populations. Thus, it will be necessary to distinguish aspects of adult migratory phenotypes for long‐distance migrating populations such as timing of freshwater entry, duration and travel time in migration corridors, tributary arrival timing, timing of arrival to spawning grounds, timing of gonadal maturation among sexes, and timing of spawning events. Validating the genetic basis and effect size for these various phenotypes will elucidate complex migration timing for interior populations of Chinook Salmon and Steelhead.
- *How do timing and patterns of juvenile outmigration relate to adult migration timing?*
  For interior populations of Chinook Salmon and Steelhead, the timing and patterns of outmigrating juveniles are highly variable compared with coastal lineage populations of each species. For example, interior ocean‐type Chinook Salmon typically exhibit subyearling outmigration but yearling smolts have often been observed (e.g., Conner et al., 2005). Further, bimodal outmigration patterns occur within multiple populations of interior stream‐type Chinook Salmon, and natural populations of inland Steelhead exhibit high variation in smoltification and outmigration timing. Variation in juvenile migratory traits has been tied to adult migratory patterns (Waples et al., 2004), suggesting a correlation and potential link between juvenile and adult life history traits that may be under genetic control.
- *Do resident populations harbor genetic variation for migration timing?*
  While Chinook Salmon rarely occur as natural resident freshwater populations (Quinn, 2018), populations of *O. mykiss* may be primarily resident due to environmental conditions (e.g., Narum, Zendt, et al., 2008), or have been excluded from anadromy by anthropogenic barriers. For these resident populations of *O. mykiss*, there is potential that they harbor important genetic variation for migration timing that could be a source for recovery of early alleles for local populations of Steelhead (e.g., Fraik et al., 2021). Additionally, freshwater populations may demonstrate variable phenotypes for spawn timing or arrival timing for spawning or spawn timing that has not been accounted for in their life history and has a genetic basis (e.g., Kannry et al., 2020).
- *Are GREB1L/ROCK1, or other genes, associated with early and late migration phenotypes in additional salmonid species?*
  While the role of GREB1L/ROCK1 has been studied extensively in Chinook Salmon and Steelhead in recent years, very little research has investigated the patterns in migration timing for other salmonid species. This is particularly intriguing for other species within the genus *Oncorhynchus* that display variation in adult migration timing. The same gene region of GREB1L/ROCK1 is conserved between Chinook Salmon and Steelhead and may also be associated with run‐timing in other salmonid species that have not yet been investigated to examine specific adult migration timing phenotypes that may be associated with outliers in this gene region (e.g., *O. nerka*, Tigano & Russello, 2022).
- *Is there structural variation in the genomic regions of Ots28 and Omy28 that can be verified with advanced sequencing technology?*
  Linkage disequilibrium is extensive within the GREB1L/ROCK1 region for both Chinook Salmon and Steelhead and evidence supports a duplication within this region for Chinook Salmon (Horn & Narum, 2023; Thompson et al., 2020). However, verification of structural variation will require long‐read sequencing that can span these complex sections of the genome as opposed to short‐read sequences that have been applied in previous studies. Multiple technologies are available to examine long‐read sequences to verify duplication or other structural variations such as inversions or alternative splice variants. This would also provide detailed maps of promoter regions that are likely located in the intergenic region and would provide insight into the regulation of either GREB1L or ROCK1, or both. Further long‐read sequencing could also enable comparison to the paralogous GREB1L/ROCK1 region to determine if gene activation or silencing is involved with migration timing.
- *What are the causal variants and functional mechanisms of GREB1L/ROCK1 genes that affect adult migration timing?*
  While patterns of association and effect size for adult migration timing have been demonstrated and validated for GREB1L/ROCK1, it is yet unknown how these genes regulate adult migration timing in Chinook Salmon and Steelhead. Studies focused on this question have the potential to utilize technology such as CRISPR to alter the underlying DNA of these genes to directly evaluate phenotypic effects, but extreme caution would be necessary to avoid the release of CRISPR‐modified fish into the natural ecosystem. Furthermore, the patterns of gene expression at GREB1L/ROCK1 have not been examined to determine how they are regulated at different life stages relative to adult migration timing.

## 5 CONCLUDING STATEMENTS

Here we synthesize the current state of science regarding genetic variation associated with adult migration timing in lineages of Steelhead and Chinook Salmon in the Columbia River Basin. The lineages of both species that migrate to the interior Columbia River display complex and distinct migration patterns from those in coastal lineages. In particular, the long migration distances for lineages in the interior region have evolved under distinct selective environments relative to coastal populations of each species (e.g., Waples et al., 2008) and demonstrate distinct patterns of association with genetic variation in the GREB1L/ROCK1 region. Further, conservation decisions need to consider the preservation of variation across the entire genome in addition to major effect genes (see Waples et al., 2022 for further discussion) to allow species to evolve under changing climate conditions (e.g., Carlson & Satterthwaite, 2011; Sturrock et al., 2020). Finally, management actions also need to consider additional complexities such as mitigation obligations in the region for each species.

While discovery of the major effect region of GREB1L/ROCK1 has led to conservation concerns for early migrating populations of Chinook Salmon and Steelhead, direct conservation actions based on the frequency of early alleles should be applied cautiously and only when adequate data is available (e.g., Kardos & Shafer, 2018). In particular, genotype‐to‐phenotype relationships need careful validation in specific populations since there are clear differences in genetic variation across lineages and populations of each species. It is not appropriate to assume that one or two markers from GREB1L/ROCK1 that are informative for a particular population are equally effective in other populations. Further, conservation actions need to consider preservation of variation across the entire genome in addition to major effect genes (see Waples et al., 2022 for further discussion).

Finally, it is apparent that extensive phenotyping at various stages of migration is a major barrier to further research in this area for Chinook Salmon and Steelhead. Novel approaches are needed to phenotype large numbers of fish with various migratory characteristics to fully examine the genetic basis of life history variation of these species. These phenotypes are necessary to combine with genomic sequencing and genotyping to enable further studies that will allow for long‐term conservation and recovery of these salmonid species.

## 7 CONFLICT OF INTEREST STATEMENT

The authors have no conflict of interest to declare.

## Supporting information


**Tables S1.** **–S2.**

## Data Availability

No new data are included in this synthesis/review. All previously published data have been made available through links in the original publications. Benefits from this research accrue through sharing the synthesis of information that has been compiled here from the results of several previous studies.
