# Supplementary material for: Genetic variation associated with adult migration timing in lineages of Steelhead and Chinook Salmon in the Columbia River
Source: Evol Appl. 2023 Dec 28;17(2):e13626. doi: 10.1111/eva.13626 (PMC10853649; doi:10.1111/eva.13626)
Supplement: Supplementary file 1 — Tables S1. –S2. [file EVA-17-e13626-s001.docx]

Supplemental Table 1: Molecular markers for candidate regions associated with run-timing in Steelhead (*Oncorhynchus mykiss*). *Polymorphic alleles in probe are listed in order of association: [early/late]. Reproduced from Willis et al. 2020; Collins et al. 2020.

| SNP Marker | Gene | Forward primer | Reverse primer | Probe |
| --- | --- | --- | --- | --- |
| Omy28_11607954 | GREB1-L | TGACACTGATCACAATGGTGAAAT | TAAACTGGAAGGAGAGAGCAAAAT | TGTGGGCTGC[A/G]AACATACTCA |
| Omy_RAD52458-17 | GREB1-L | ACGTGTCCCTGAGGATGGTA | AGCTCTAGGTCTGGGTCCTG | ATGGCCC[C/A][CT]AAGAACCC |
| Omy_GREB1_05 | GREB1-L | TGGGCAGATATGGAAGAACGG | ACCTTCTAAATGGCCTCTGTGT | CGGTGGCTC[T/G]C |
| Omy28_11625241 | GREB1-L | CAACATTTAGGGAGAGGTTGCTAT | ATCATCAAGTTTGCCTACGACAC | CCTCCTCCCT[A/G]TGGTTGTCTC |
| Omy28_11632591 | GREB1-L | GTAGAGGCCAAAGGCTTGAG | TGCTCTTATTACCTTCCAGACTCC | TGAGAA[G/A]AACACAGAGG |
| Omy_GREB1_09 | GREB1-L | CCAGTGGCAACCTCAGGTAG | GACTCCAGTCACCCAAGTCA | TCAA[T/G]GGAGA |
| Omy28_11658853 | intergenic | CAACATATGACCACTCGAAAACTC | ATTAATCACACCGTGAGACTCCTC | TGGTACAGAC[A/C]CGCACTAGCA |
| Omy28_11667578 | intergenic | ACAGTAAACCCATTCAGGCATAGT | TTATCCTCTCAATCCACATCAAGA | GTATTGATCC[T/C]GTGGGAGACA |
| Omy_RAD47080-54 | intergenic | TCAAAACCTGCAGGACTTGGA | TGGTTATATCTACAGTACAGTTCGT | TGCAAG[A/G]CTTAAAACGA |
| Omy28_11671116 | intergenic | AATTTCCCCAAATTTGAAACTCTT | GTGTACATTGTCAGGCAGAAACAT | CTGGTGAGAA[C/T]AGGAATTACC |
| Omy28_11676622 | intergenic | CGAATGCACTGTAGCTCATTCTAA | GCAGTAGAATGTCTCGCAAATACA | ACATGTCATT[T/G]ATTGTTATCT |
| Omy28_11683204 | intergenic | CAAGAAAGAAACAGATGTTGTCCA | TTGTGACTCAAATCTGCAACCTAT | ATGTAAAAAA[G/T]GGCAGAAAA |
| Omy28_11773194 | ROCK1-L | AGTTTGACACCCCTGTACTAGAGC | GTCTAACAAGCTCTGGGTGATTTA | GCAATTTTTT[T/A]AAATTACCGC |

Supplemental Table 2: Molecular markers for candidate regions associated with run-timing in Chinook Salmon (*Oncorhynchus tshawytscha*). *Polymorphic alleles are listed in order of association: early/late. Reproduced from Koch and Narum 2020; Willis et al. 2021.

| SNP Marker | Gene | Forward primer | Reverse primer | Probe |
| --- | --- | --- | --- | --- |
| Ots28_11023212 | greb1L | AGAAAGCCATCATCATGAGACC | ACAAACAAACAAAAATGGTCAGAA | AACGTGACAC[A/G]AT |
| Ots28_11025336 | greb1L | TGCAATATAGAACAAATCCGAAAA | AATAACCCTTGGCTTCACATACAT | CAATGAAGTT[A/C]ATTTAATTGG |
| Ots28_11033282 | greb1L | GGCTTTCTGATGATCTTGAACTTT | AGTGTGAGAGAGAGGAAGTCCCTA | TAAAAATG[G/A]TTGATATGTA |
| Ots28_11062192 | greb1L | AGATGATATGGATTTGCTGTGTGT | TTGAACATAACGATCAGAGAAAGA | TTCTCAAGTC[C/G]TACTCAACTG |
| Ots28_11070757 | greb1L | TTTTGGAACCCTTTTTACTACGAG | ACATCAGTATAGCAGAGGAGAGGG | ACCCATGAAT[A/G]AGGACGAGAG |
| Ots28_11071377 | intergenic | ATTTGCTGTGTGTGGAGTGAAT | GTAGTGACAGATGCTCTTGGAGG | CATCTTAGCC[T/C]CTCTGACCCC |
| Ots28_11072994 | intergenic | GGGAGACTTAAAACAACCTCAAAA | ACCTGCAACCTTCTATTCAACAGT | CCATATGTCG[C/T]TTGT |
| Ots28_11073102 | intergenic | GGTGAGCCATTCATAACAATCTT | TGTTATCCTGGATCATTCAAGAGA | ACATTACTTT[T/A]CAAAAATATT |
| Ots28_11073668 | intergenic | CCTAAGAGGAGACGAGCATTACAG | GGTAAATCAACATATGACCACTCG | TACAGTTTCC[T/A]GTCTGA |
| Ots28_11075348 | intergenic | CATTTCAAAATTAGGAGGTTAGGG | AGATGAGAGCTGTGGCCTGT | GTGTGAAAGG[G/A]GAGAAGGGCT |
| Ots28_11075712 | intergenic | GCTTAAACAGCTGCTATTAGGACA | TAAGGATTTGTTGCCAGCTCTAAT | GAAAACTCTG[C/T]CCTG |
| Ots28_11077016 | intergenic | AAAATATGTGCAACATCCAATGTC | ACACAAGCTGGCTGAAGCTAAT | GTCAAACCAA[C/T]TTTGCCAAGG |
| Ots28_11077172 | intergenic | GTTTTGCCAGAGAGAATGTACAAA | TAGTGGTTAGAGCATTGGACTAGC | ACACACACAA[G/A]AGACACCCAC |
| Ots28_11077576 | intergenic | TGTGCGGAATTACTGATAATTGAC | GCTCTGCATTTTACAACACTGCT | GAAGGCC[A/G]AATAAAATTG |
| Ots28_11095755 | intergenic | CCAATGGTGATTTTAGAACCATTAC | AAAACAGAGTATGGATCAACAGCA | AG[T/A]GTTGAATGGC |
| Ots28_11143508 | intergenic | ACCTTTTAGCCAGTGACAACATTT | ATGCAAGAAACTCTCGACGATAG | TTCAC[A/G]TACGGCCCAT |
| Ots28_11160599 | rock1 | GTGCATATTTTACGTGGTTGAAGT | ATTCCATTTCACCCATATGAATTT | CTCTCTGCTT[T/G]CGTT |
| Ots28_11164637 | rock1 | TGATTTGACTTTTTGTGGTGTTTT | GTTCCAATCTGTTTTTGCTCTCTT | CTGGCGGGGT[A/C]TGGG |
| Ots28_11186543 | rock1 | GGCTTGCCTTTAGATAGAATCTTG | AAATCTCACAAGTCCAAAAACAAA | AAAGCTGATT[T/A]AAAA |
| Ots28_11201129 | rock1 | TGCGAGATTTATCTACTTGTCCAG | GGTAGTTTTGTACGCAATTGCTAA | ACTGAAGGAA[G/T]TTAAC |
| Ots28_11202190 | rock1 | GCTAAATGTAAATCGAGTGGCTGT | TACATGGGTCCTCTCAGTGTTCTA | CAAAAGTCTG[C/T]ATTTTCAAAA |
| Ots28_11202400 | rock1 | CCCTCCAAAAAGAAAACATTTGAT | AAATTGGCTAATCAAACACTGGTT | GACACACTCA[T/C]GA |
| Ots28_11202863 | rock1 | GAGGATGGATGAGACTTTTCAGAT | GCTCTTTACCGGGTTTATATGAAG | ATAAAAAATT[A/C]TGCGTGAATG |
| Ots28_11205423 | rock1 | TTAAATCACCCAGAGCTTGTTAGA | ACCTGACCTAGATAACAACCACAA | CCTGCACAC[G/A]TGTCAAACCG |
| Ots28_11205993 | rock1 | GCTGCTATTTCCGACCTTACAATA | ATCAAGACAAAACACTCACCAGAA | G[T/C]TATTAAAAGG |
| Ots28_11206740 | rock1 | ACTTTGAGGACTTACTCCTGTCCT | CTGGAGAAAGACAAGATGATGATC | CCTTCCCTCC[C/T]AGGGCAACGT |
| Ots28_11207428 | rock1 | TATACCTTTGTAGCATCCCTCTCC | CATATAAAGTGGACAGCGTTTGAC | GTTGGGAGCG[G/T]CCCAAAATGG |
| Ots28_11210919 | rock1 | AGTGCTCCATGCTGGAGTTT | GATGAAGCAGAAGGAGAGGCT | GACCT[T/C]AAGCAGTCAG |
